# Learning apps at home prepare children for school

**DOI:** 10.1111/cdev.14184

**Published:** 2024-10-24

**Authors:** Frank Niklas, Efsun Birtwistle, Anna Mues, Astrid Wirth

**Affiliations:** ^1^ Department of Psychology University of Munich (LMU) Munich Germany; ^2^ University of Nottingham Nottingham UK; ^3^ University of Vienna Vienna Austria

## Abstract

The usage of high‐quality learning applications (apps) at home may increase children's mathematical and literacy competencies. This approach was tested in a family intervention study. Intervention families (*n* = 302) in two German cohorts (*N* = 500; *M* (SD)_age_ = 61.0 (4.6) months; *n*_*♀*_ = 302) received tablets with newly developed learning apps focusing either on mathematical or literacy learning for every‐day usage at home across half a kindergarten year. Compared with two control groups with and without tablets, children in the intervention groups significantly enhanced their specific competencies (*η* ^2^ = .03–.10). Higher app usage was associated with greater gains (Δ*R* ^2^ = .01–.02). Consequently, an easy‐to‐apply app‐based intervention supports children's development of cognitive competencies and helps prepare them for school.

**Abbreviations**
AWST‐R: Aktiver Wortschatztest für 3‐ bis 5‐jährige Kinder—Revision (active vocabulary test for ‐3‐5‐year old children‐ revision)
CMMS: Columbia Mental Maturity Scale
EF: executive function
EuLe 4–5: Erzähl‐ und Lesekompetenzen erfassen bei 4‐ bis 5‐jährigen Kindern (assessment of narrative and reading competencies of 4‐ to 5‐year‐old children)
HLE: home learning environment
ITT: intent‐to‐treat
PA: phonological awareness
PPVT: Peabody Picture Vocabulary Test
SES: socioeconomic status
SETK 3–5: Sprachentwicklungstest für drei‐ bis fünfjährige Kinder (linguistic development test for 3‐5‐year‐old children)
WVT: Würzburger Vorschultest (Würzburg preschool test)

In addition to formal educational contexts, the home learning environment (HLE) and thus the family is the most important setting that can support young children's competencies development (Niklas & Schneider, 2017a; Sénéchal & LeFevre, 2002). Within the HLE, high‐quality literacy and numeracy resources and interactions support the development of children's literacy and mathematical competencies (Aikens & Barbarin, 2008; Anders et al., 2012; Ramani & Siegler, 2008; Sénéchal & LeFevre, 2002; Silinskas et al., 2020). The quality of the HLE is closely associated with a family's socioeconomic status (SES), and not all families are able to support their children's learning adequately (Wirth et al., 2023). In particular, high‐SES families are more likely to enrich their children's learning with ample resources and frequent, high‐quality interactions driven by their high income, high educational background, and more prestigious occupations (Aikens & Barbarin, 2008).

Early interventions have the potential to make a difference for children's competencies development (Heckman, 2006) and previous studies showed that family literacy and numeracy interventions may successfully support children's learning (e.g., Mol et al., 2008; Nelson et al., 2024; Petersen‐Brown et al., 2024). However, only a few studies applied interventions via digital media and mobile applications (apps) that are currently available and used in households (Berkowitz et al., 2015). In addition, app intervention studies were often conducted in laboratory settings (Maertens et al., 2016) or focused only on one specific learning target (Berkowitz et al., 2015). Therefore, in our experimental field study, we tried to address a perceived gap by analyzing children's literacy and mathematical competencies and the potential impact of an app‐based family intervention on this development while controlling for important child (e.g., intelligence) and family characteristics (e.g., SES).

## Development of mathematical and literacy competencies in kindergarten children

Although the formal learning of mathematics, reading, and spelling starts when children enter primary school, children acquire specific competencies that prepare them for later learning long before school entry (e.g., Lehrl et al., 2020). For instance, arithmetic achievement in first grade is predicted by kindergarten children's early counting abilities (Stock et al., 2009) and their ability to link quantities to number words (Krajewski & Schneider, 2009). Nguyen et al. (2016) showed that such early numeracy abilities are the strongest predictors of later mathematical achievement in school. Similarly, later reading comprehension is predicted by young children's early vocabulary (Joshi, 2005) and phonological awareness (PA; Melby‐Lervåg, 2012). Consequently, numeracy and literacy precursor abilities enable and support children's later learning of mathematical and literacy competencies (e.g., Krajewski & Schneider, 2009; Torppa et al., 2007) and potentially provide children a head start at the beginning of school and beyond for primary school. In the following, we provide a short overview of the development of relevant early literacy and mathematical competencies.

Oral language skills such as vocabulary and code‐related skills such as letter knowledge and PA are interrelated according to the emergent literacy model (Whitehurst & Lonigan, 1998). For instance, PA refers to children's ability to identify rhyming words and onset sounds of words and thus phonemes (Melby‐Lervåg, 2012). Both, the early ability to use or manipulate language expressively and the ability to understand language are important prerequisites for children's later literacy development. Although language complexity differs across different languages (Seymour et al., 2003), linguistic precursors that have been identified in English language contexts also predict children's literacy development in German language contexts (Ennemoser et al., 2012; cf. Niklas & Schneider, 2015).

In her model of the early development of quantity‐number competencies, Krajewski (Krajewski & Schneider, 2009) identified basic numerical skills such as counting and discrimination of quantities and the later quantity to number word linkage to be important steps in the transition from a procedural to an increasingly conceptual understanding of number words and arithmetic. In addition, these abilities measured in preschool were found to be better predictors of mathematical competencies in Grade 4 than intelligence, number naming speed or SES. Therefore, children's early numeracy abilities seem to be the basis for higher order mathematical thinking and competencies (Jordan et al., 2006).

According to Ricken et al. (2013), children have to acquire various mathematical abilities such as counting and an understanding of cardinality to master more advanced mathematical tasks. Indeed, young children's level of mathematical knowledge proved to be an effective predictor of later mathematical achievement (Fritz et al., 2018). In addition to specific precursors, other cognitive abilities such as intelligence and child characteristics such as age and sex should be considered when analyzing the development of child competencies and the support of these competencies via interventions (Schneider et al., 2014).

## Interventions to support children's competencies development

Interventions to support children's competencies development should ideally start as early as possible. Early interventions are more cost‐effective compared to later interventions, because they address issues at a stage when potential problems (e.g. learning difficulties) can be more readily mitigated or even prevented, thereby reducing the need for more extensive and costly treatments or support in the future (Heckman, 2006). Indeed, successful intervention studies have been conducted to train young children's early numeracy and literacy abilities (e.g., Mol et al., 2008; Nelson et al., 2024; Petersen‐Brown et al., 2024).

The HLE is the context in which young children usually acquire important competencies before they start school (Anders et al., 2012; Sénéchal & LeFevre, 2002; Silinskas et al., 2020). Consequently, the HLE is a good target for interventions to support the development of children's early competencies. Meta‐analyses in this context show medium to large effects for such family interventions (Nelson et al., 2024; Petersen‐Brown et al., 2024).

For instance, children's numerical abilities were improved by a non‐intensive intervention study, in which parents attended a parent evening, that provided them with information about the importance of the home numeracy environment (Niklas et al., 2016). In addition, parents and their child were observed while they were playing a dice game that the child was presented as a gift and parents received feedback on how to assist their child's learning during this game. The results suggest that interventions in the HLE may induce positive numeracy learning outcomes (e.g., counting, number values). Furthermore, in a recent study by Purpura et al. (2021), parents were instructed to read picture books with embedded mathematical language content to their preschool children at home. Twelve readings of these books lead to significant positive effects on mathematical language and numeracy compared to a control group with picture books without mathematical content.

In another study by Niklas and Schneider (2017b), families in the intervention group received a children's book to keep and were provided with information about the home literacy environment and were given feedback on shared reading. Intervention children's vocabulary and PA improved significantly compared to children's skills in the control group. Furthermore, a meta‐analysis by de Bondt et al. (2020) examined the effects of book giveaway programs on the HLE and children's competencies. Their results show that distributing or sending books and information flyers about shared reading with children to families supports children's home literacy environment resulting in more reading interest and better literacy related competencies. These studies indicate that by providing additional learning materials and feedback on their usage, primary caregivers can support children's precursors skills.

In addition to such analogue intervention approaches, novel technological developments also enable the usage of digital media for family interventions. For instance, Wood et al. (2020) were able to show that both a traditional text reading workshop for parents of kindergartners and a workshop that additionally focused on computer‐assisted learning opportunities were both successful in improving language and literacy skills of children. The second workshop included additional content such as assessing early reading software programs for content and age/skill appropriateness and evaluating children's reading software design. Furthermore, easy‐to‐apply methods such as simply installing learning apps from app stores for their kids may be attractive for families of young children (Judge et al., 2015).

## App‐based interventions

Given the prominence of digital media in many households (e.g., Chaudron et al., 2018), most children are in daily contact with these media and digital apps from an early age on (Papadakis et al., 2018). Although research shows that long screen times can have a detrimental impact on children's behavior and learning (e.g. Eirich et al., 2022; Fang et al., 2019; Janssen et al., 2020), high‐quality digital media may support children's learning (e.g. Berkowitz et al., 2015; Neumann & Neumann, 2017). In addition to the usage just for entertainment, digital media offer the opportunity to promote children's early competencies development and open up the possibility of tackling traditional problems in education such as access to education for all children and their families (Papadakis et al., 2018) and improving the quality of learning environments (Lee & Choi, 2020).

The use of apps as an educational tool becomes more and more established in the early childhood context, for example in kindergartens and schools, as well as in the HLE (Hirsh‐Pasek et al., 2015). However, only few apps on the market have ever been evaluated concerning their quality and effectivity, and even the most downloaded apps on the market are, on average, rated as low quality (Meyer et al., 2021).

In a seminal work by Hirsh‐Pasek et al. (2015), the “four pillars of learning” framework was introduced to define the learning impact of educational apps. This framework is based on general principles of children's learning and suggests that high‐quality apps support active, engaged, meaningful and social interactive learning and foster a specific educational learning goal.

“Active” app‐based learning is a “minds‐on” learning while using the app, whereas “engaged” refers to the avoidance of distracting elements and thus enabling children to remain focused during the app usage. A “meaningful” app enables children to build upon their existing knowledge and expand their concepts, whereas “socially interactive” refers to greater app‐based learning success, if the app is used together with more knowledgeable persons, in collaborative situations, and, in general, in a social context. Indeed, Kim et al. (2021) report an effect size of +0.31 on children's literacy and mathematical skills for children from preschool to Grade 3 for the use of educational apps in their meta‐analysis (see also Neumann & Neumann, 2017).

Consequently, tablet‐based family interventions have the potential to support the competencies' development of all children at low‐cost, and offer to be a high‐benefit method. Indeed, empirical evidence of recent years points to positive correlations between the use of touchscreen‐based devices, digital games and children's numeracy and literacy skills in various studies (Barnyak & McNelly, 2015; Berkowitz et al., 2015; Dejonckheere et al., 2015; Moyer‐Packenham et al., 2016; Neumann & Neumann, 2017; Papadakis et al., 2018, 2021; Schacter & Jo, 2017; Schaeffer et al., 2018; Schenke et al., 2020; Shamir et al., 2012). Here, interventions that used multi‐touch‐based devices supported children's learning through the use of apps (e.g., Outhwaite et al., 2019), even for children who had previously no experience with digital devices (Lee & Choi, 2020).

For instance, Berkowitz et al. (2015) used an iPad app to deliver short numerical story problems to first graders and their parents. Compared with a reading control group, more intense usage of the mathematical learning app was associated with greater gains in mathematical competencies. Similarly, the use of high‐quality e‐books can enhance young children's emergent literacy such as vocabulary, print awareness, and PA (Shamir et al., 2012).

## Research focus

Previous research has shown that children can profit from family intervention studies (e.g., Niklas & Schneider, 2017b) and that greater usage of high‐quality learning apps can enhance their competencies (e.g., Berkowitz et al., 2015; Maertens et al., 2016). However, some limitations apply to previous studies. For instance, they were (1) not ecologically valid as they were conducted in laboratory settings, (2) focused only on literacy or numeracy learning, (3) did not assess intervention fidelity, (4) used single apps, or (5) did not control relevant child and family characteristics. With our approach, we overcame these limitations in a large‐scale family intervention study and took previous research one step further in an experimental family study design.

We used data from the app‐based intervention study “Learning4Kids” conducted in Germany (see Niklas et al., 2020, 2022). Here, we used the first two measurement points to check whether an app‐based intervention had any impact on the development of children's outcomes. Children in the study were randomly assigned to either a literacy intervention, a numeracy intervention, a tablet‐control, or a business‐as‐usual control group without tablets. In our confirmatory research approach, we expected children in the literacy intervention group to improve their literacy competencies (Hyp. 1a) and children in the numeracy intervention group to improve their mathematical competencies (Hyp. 1b) significantly compared to their peers in the control groups (Berkowitz et al., 2015; Niklas & Schneider, 2017b; Shamir et al., 2012). Furthermore, we expected longer app usage to be associated with greater gains (Hyp. 2a and 2b; Berkowitz et al., 2015). Finally, we were interested in whether apps with a specific focus on either number knowledge, counting, letter knowledge, or PA led to gains in these specific competencies (Hyp. 3a–3d; Barnyak & McNelly, 2015; Berkowitz et al., 2015; Dejonckheere et al., 2015; Moyer‐Packenham et al., 2016; Neumann & Neumann, 2017; Papadakis et al., 2018, 2021; Schacter & Jo, 2017; Schaeffer et al., 2018; Schenke et al., 2020; Shamir et al., 2012). All of these hypotheses were already included in the original project proposal and thus established before the study was conducted, making them a priori hypotheses.

## METHOD

### Sample

Our total sample consisted of 500 children from 329 German kindergartens in Munich and its surroundings and corresponded exactly to the number of participants we aimed at according to power analysis and considering potential drop‐outs. The children in our study were recruited in two cohorts. The first cohort (*N* = 190 children) started the assessments in summer 2020 (for all details about this cohort, see Niklas et al., 2020). The second cohort (*N* = 310 children) was recruited about 9 months later and the children were about 4 months younger than the children in cohort 1 at the start of their first assessments (for all details about this cohort, refer to Niklas et al., 2022).

In the complete sample, 69.1% of the families had a total monthly household income of at least 3.419 € and in 65.1% of the households at least one of the main caregivers held a university degree. In addition, the Magnitude Prestige Scale (Wegener, 1988) was used to rate the socially recognized prestige of parental occupation. The scale ranges from 20.00 (unskilled laborer) to 186.80 (physician) and this range was also found in our sample. The highest average prestige score in our sample was *M* = 101.0 (SD = 36.5), which indicates an above‐average SES compared with other German samples (Niklas et al., 2020; Novita et al., 2022). The SES of the family was calculated as the mean score of the standardized scores for parental education, parental income, and parental occupational prestige.

Furthermore, 42.2% of the sample had a migration background, i.e., one or both parents were born outside of Germany. We focused on children in the second half of their second‐to‐last year of kindergarten, that is, when children were about 5 years old. Children ranged in age from 51 to 75 months at t1, with an average age of *M* = 60.96 months (SD = 4.61) and 255 children were girls.

Between t1 and t2, five children dropped out as their families moved to another city. No significant differences between these five children and their study peers were found for the study variables (*p* > .05) with the exception of passive letter knowledge and grammar, in which the children who dropped out were significantly better than the children remaining in the sample.

### Procedure

In cohort 1, 114 families were recruited via the kindergartens attended by the study child in the beginning of 2020. Kindergartens in Munich districts with higher rates of unemployment and low income and with a higher ratio of families with a migration background were approached to include families with a wider range of family SES. The project was introduced and explained to kindergarten directors and educators via telephone calls or in person. To make sure that families understood the contents and the procedure of the study clearly, plain language statements and consent forms were provided to parents, also in the most commonly used foreign languages of the families, if needed. Additional families were contacted and recruited via a social media campaign. Finally, five families were recruited by word of mouth and leaflets distributed in the city.

In cohort 2, again a couple of families were recruited via leaflets; however, almost all families were approached via a postal letter sent to their home in the end of 2020 and the beginning of 2021. The contact details of Munich families with children in the target age group were provided by the Department of Public Order. Interested families were asked to get in contact with the project team and were then provided with plain language statements and consent forms. We stopped the recruitment once the target number of 500 families was reached.

All children were randomly assigned to one of four conditions (literacy intervention, numeracy intervention, tablet‐control, control group without tablet). The children were tested before and after the intervention phase at home apart from a few families who decided to visit the university for the assessment and who were tested in quiet rooms. The assessment was comprised of two parts. In the first part, the assessments consisted of 30–40 min standardized child testing, followed by a break in which one main caregiver played a dice game with the child and read a book to them. In the second part, children were assessed again for about 30–40 min with standardized tests and they received small gifts. Families in the tablet groups then received their tablet to keep and use for 168 days during the intervention phase (see below) after which the t2 assessments took place for all children.

### Test instruments

In the following, the test instruments to assess children's mathematical and literacy competencies and intelligence are described (for all test references and more information see Niklas et al., 2020, 2022).

#### Mathematical competencies

Altogether six standardized mathematical subtests for kindergarten children addressing children's number (symbol) knowledge, counting, calculation, and comparing of numbers and amounts were applied. The “Mathematik‐ und Rechenkonzepte im Vorschulalter—Screening” (MARKO‐S) assessed cardinality, numbers knowledge, number division, ordinal number bars as well as inclusion and relations. Based on different subtests from the “Würzburger Vorschultest” (WVT), children's number sequence forwards, number sequence backwards, knowledge of numerical representation and number symbol knowledge were assessed. An adapted version of the calculation task from the “Test mathematischer Basiskompetenzen im Kindergartenalter” (MBK‐0) was used to test children's early arithmetic skills. Reliability of the total score in mathematical competencies for the total sample based on the sum score of these subtests was *α*_t1/t2_ = .90/.91 (Ω_t1/t2_ = .88/.90). For the analyses, the mean of the standardized sum scores of the subtests was used.

##### Literacy competencies

Children's vocabulary, PA, early literacy, grammar, and letter knowledge were assessed with various standardized tests. Here, the “Aktiver Wortschatztest für 3‐ bis 5‐jährige Kinder—Revision” (AWST‐R) assessed children's active vocabulary and the German version of the “Peabody Picture Vocabulary Test” (PPVT) assessed children's passive vocabulary. Furthermore, PA was tested with a rhyming task and an initial sound analysis task from the WVT. Two additional subtests of the WVT assessed children's active and passive letter knowledge. In addition, in a grammar test from the “Sprachentwicklungstest für drei‐ bis fünfjährige Kinder” (SETK 3–5), children's ability to build plural forms of words and non‐words was tested. Finally, the subtest “Schriftwissen” from the test “Erzähl‐ und Lesekompetenzen erfassen bei 4‐ bis 5‐jährigen Kindern” (EuLe 4–5) was used to assess general early literacy knowledge (e.g., “Please show me, where you start reading in this book”). Reliability of the total score in literacy competencies for the total sample based on the sum score of these subtests was *α*_t1/t2_ = .84/.85 (Ω_t1/t2_ = .77/.72). For the analyses, the mean of the standardized sum scores of the subtests were used.

#### Intelligence

Children's intelligence was measured with the Columbia Mental Maturity Scale (CMMS) which is a short non‐verbal intelligence test for young children. Here, children had to identify the one odd picture in an array of three to five pictures (e.g., the fork among several spoons). In total, the test comprises three example items which are used to introduce the test procedure and the task for the children and 57 test items. Reported reliability and prognostic validity of the CMMS are good (see Esser, 2002; split‐half reliability ranging between .92 and .96).

### Intervention

The intervention in Learning4Kids used a tablet‐based approach (see Niklas et al., 2020). In the intervention, mainly new learning apps were designed and developed (*N* = 40) in addition to the identification and usage of some already existing learning apps (*N* = 8) (for an overview refer to Table S3 in Appendix S1). The recommendations of Hirsh‐Pasek et al. (2015) were considered as quality criteria for the learning apps, so that our apps should be suited to promote efficient learning of kindergarten children at home. Indeed, independent experts rated 18 of our apps according to this framework and the apps used in our study received significantly higher ratings than conventional educational apps for children, which are publicly available in the app stores (Wirth et al., 2024; Meyer et al., 2021).

In our randomized‐control‐group design, children and their families were assigned to either one of two intervention groups or one of two control groups (one control group with tablets and the other one without tablets). The two intervention groups received tablets with literacy and numeracy apps, whereas the tablet‐control group received tablets with control apps that did not focus on literacy and numeracy, but more on general cognitive abilities such as executive functions (EFs). Children and families were familiarized with the tablet and the apps that were available at the beginning of the study during the first family visit. The project team recommended families to use the tablet computer regularly during the intervention phase and to let the children play the apps as their main caregivers saw fit. We further recommended to use the tablet no longer than a maximum of 30 min per day.

If possible, the apps were developed as similarly as possible, with the same games for all three groups, but with different stimuli depending on the group (examples see below and refer to Niklas et al., 2020). For instance, a memory game app was provided to all groups. In the literacy app, memory cards were presented with letters and in combination with animals whose names start with a matching letter sound; in the numeracy app, numerical values in different forms such as dots on dices, fingers of a hand, lines, or Arabic numerals were used, and in the control app a colors memory game was provided. Other examples were apps about drawing (letters, numbers and colors), a snake & ladders game (with letters, numbers and colors), and a finding the pair game (rhymes, number representations and colors). With this approach, we tried to keep the input as similar as possible for all groups. However, this was not possible for all apps, as some apps focused on very specific content such as “Find the vowels” and “Sentence understanding” (both literacy) and “Measurement learning” and “Learn the clock” (both numeracy).

Literacy apps focused on linguistic development with activities such as letter learning, letter drawing and sorting, rhyming, PA, and word and sentence understanding. Mathematical apps included numerical activities such as number learning, number drawing and sorting, counting, measurement, and learning the clock. Control apps included games based on domain general skills such as sorting, drawing, concentrating, and shape and color recognition as well as puzzle and games fostering hand‐eye coordination. To keep children motivated for the complete intervention phase, all tablet groups started with some apps and each month new apps were installed to their tablets (see Niklas et al., 2020; Table S3). Here, mostly easier apps and apps training more basic competencies (e.g., letter and number knowledge) were available from the start of the intervention phase und more difficult apps and apps training more advanced competencies (e.g. sound identification within words and learning the clock) were added later.

Children's learning with the apps was supported by several features of these apps. For instance, step‐by‐step verbal instructions, a level structure with increasing difficulty, and built‐avatars acted as scaffolds for the app usage. Furthermore, verbal and visual feedback on the performance and reward systems were provided to support the learning, motivation, and engagement of children. In addition, usability was high as mainly easy drag and drop and tapping responses were required.

### App usage

The app usage was assessed via mobile sensing (for detailed information about mobile sensing and its application in Learning4Kids, refer to Birtwistle et al., 2022). To this aim, the Phone Study App measured the exact time when a learning app was played in the foreground of the display of the study tablet and added this time up for the complete intervention phase (i.e., app usage time). Consequently, we were able to assess the exact usage time of each tablet and each app. For the mathematical and the literacy intervention group, the total app usage time was calculated and used in the analyses.

In addition, the usage time of some apps that focused on the same competencies were combined to assess a specific total learning time for these competencies. For example, all mathematical apps focusing on number (symbol) knowledge were combined (e.g., “Finding pairs (Numbers)”, “Build a number rocket”, “Memory (Numbers)”) and all apps focusing on counting were combined (e.g., “Snakes & Ladders (Numbers)”, “Mathemarmite”, “Collecting nuts (Numbers)”). Similarly, the usage time of all literacy apps focusing on letter knowledge were summed up (e.g., “Memory (Letters)”, “Snakes & Ladders (Letters)”, “Letter sorting”) as were all literacy apps focusing on PA (e.g., “Finding pairs (Rhymes)”, “Find the vowels”, “Initial letter sounds”).

Due to technical problems with some tablets, or problems in the firewall settings in some families, and some problems with the Phone Study App, the usage times could not be recorded for 10 families in the mathematical intervention group and for 14 families in the literacy intervention group. A comparison between the families with and without usage data showed no significant differences for the literacy intervention group (*p* > .05) with the exception of migration background and the gain in literacy competencies between t1 and t2. A greater ratio of families with a migration background was among the 14 families with no usage data and children in these families showed a greater gain in literacy competencies between t1 and t2, thus indicating that the intervention effects would have been even larger, if these families could have been included in the per protocol analyses. Families in the mathematical intervention group without usage data had a significantly higher SES score. No other significant difference was found (*p* > .05).

### Overview of statistical analyses

As each child was assessed in the family context and as the 500 children in our sample were distributed across 329 German kindergartens (mostly only one or two children attended the same kindergartens and for 19 children the information about kindergarten attendance was missing), analyses were conducted at the individual level only.

First, we analyzed intervention effects in a global test with the total sample in an intent‐to‐treat (ITT) analysis that allowed to test potential effects without considering actual app usage—and thus ruled out factors such as motivation and interest. For the numeracy intervention group (151 children), data of children in the literacy intervention group were combined with the data of children in the tablet‐control group (resulting in a tablet‐control group with 249 children). Similarly, the numeracy intervention group was combined with the tablet‐control group and acted as tablet‐control group (with again 249 children) for children in the literacy intervention group. Repeated‐measures analyses of variance were conducted with child age, sex, and intelligence and family SES and migration background as control variables (hypotheses 1a and 1b). In addition, exploratory analyses were conducted to check the robustness of the results for both cohorts, different outcome measures, and for the comparison of all four groups.

In a second step, we tested whether a longer usage of the provided apps resulted in a greater competencies' gain. Here, regression analyses were conducted to predict either literacy or numeracy outcomes at t2. In model 1, the competencies at t1 were controlled, before in model 2 child and family characteristics were controlled for. In the final model, the total app usage time was added to the model (hypotheses 2a and 2b).

Finally, we used regression analyses to predict specific outcomes by the apps that trained these outcomes while controlling for same variables mentioned above (hypotheses 3a–3d).

## RESULTS

### Descriptive statistics and correlational analyses

Table 1 shows the descriptive statistics for the child and family variables and the outcome measures at t1 and t2 across the four groups and for the total sample. No significant differences in any of the study variables at t1 were found and the groups were thus comparable before the intervention. At t2 and thus after the intervention, significant differences were found for number knowledge (in favor of the numeracy intervention group) and for letter knowledge and letter sound identification (in favor of the literacy intervention group).

**TABLE 1:** Descriptive statistics of the study variables across the intervention and control groups.

Variable	Literacy		Numeracy		Tablet‐control		Control group		Total	
	*M*	SD	*M*	SD	*M*	SD	*M*	SD	*M*	SD
Sex (1 = girls; 0 = boys)	0.50	0.50	0.53	0.50	0.54	0.50	0.49	0.50	0.51	0.50
Age	60.77	4.43	60.99	4.50	60.92	4.67	61.26	5.03	60.96	4.61
SES	0.06	0.79	−0.01	0.86	−0.02	0.80	−0.08	0.91	−0.01	0.84
Migration background	0.40	0.49	0.46	0.50	0.40	0.49	0.42	0.50	0.42	0.49
Intelligence	51.70	6.88	52.35	4.34	51.40	8.34	52.47	3.77	51.99	6.03
MARKO‐S t1	12.41	4.19	12.06	4.02	12.52	4.17	12.12	3.73	12.27	4.04
Counting backwards t1	3.23	2.09	3.25	2.02	3.55	2.13	3.20	1.96	3.29	2.05
Counting forwards t1	1.11	1.32	0.97	1.18	1.27	1.32	1.09	1.17	1.09	1.25
Predecessors and successors t1	1.80	1.95	1.73	2.03	1.77	1.88	1.61	1.64	1.73	1.90
Number (symbol) knowledge t1	3.94	2.96	3.99	2.83	4.04	2.89	3.96	2.80	3.98	2.87
Calculating t1	3.88	2.31	3.60	2.15	3.76	2.30	3.51	1.99	3.70	2.19
Letter sound identification t1	3.00	2.86	3.18	2.87	3.19	2.60	2.92	2.73	3.07	2.78
Rhyming task t1	4.06	2.40	4.11	2.38	3.96	2.25	3.99	2.38	4.04	2.35
Active vocabulary t1	7.16	3.83	7.21	3.72	6.85	4.16	7.46	3.70	7.17	3.83
Passive letter knowledge t1	5.63	2.73	5.82	2.91	5.79	2.73	5.42	2.74	5.68	2.79
Active letter knowledge t1	3.65	2.93	3.86	3.43	4.00	3.32	3.60	3.04	3.77	3.18
Passive vocabulary t1	65.87	24.1	62.79	24.42	64.29	24.74	64.77	21.68	64.41	23.82
Grammar t1	5.24	3.05	5.10	2.82	5.57	2.87	5.13	2.79	5.24	2.89
Early literacy t1	7.31	3.40	7.37	3.20	7.66	3.16	7.68	2.91	7.47	3.20
MARKO‐S t2	14.27	3.67	14.53	3.71	14.59	4.08	13.99	3.49	14.35	3.73
Counting backwards t2	4.38	2.27	4.39	2.26	4.46	2.36	4.10	2.22	4.35	2.27
Counting forwards t2	1.82	1.59	1.97	1.58	1.96	1.61	1.74	1.45	1.88	1.56
Predecessors and successors t2	2.82	2.34	3.05	2.23	2.85	2.43	2.65	2.26	2.86	2.31
Number (symbol) knowledge t2	5.26	2.89	5.96	2.77	5.27	3.10	4.85	2.96	5.39	2.93
Calculating t2	4.97	1.99	4.83	2.08	4.89	2.16	4.60	2.11	4.84	2.08
Letter sound identification t2	4.88	2.58	3.84	2.90	4.35	2.94	3.61	2.74	4.21	2.82
Rhyming task t2	5.32	2.18	4.85	2.20	4.84	2.31	5.06	2.13	5.03	2.20
Active vocabulary t2	8.68	3.94	8.46	4.05	8.47	4.14	8.25	3.82	8.49	3.98
Passive letter knowledge t2	7.90	2.27	6.53	2.87	6.35	2.86	6.51	2.60	6.90	2.72
Active letter knowledge t2	6.37	3.01	4.82	3.37	4.89	3.31	4.43	3.25	5.22	3.31
Passive vocabulary t2	73.97	21.01	71.94	22.42	69.86	26.94	72.20	20.52	72.19	22.60
Grammar t2	6.05	2.99	5.90	2.87	6.41	2.91	5.82	2.78	6.03	2.90
Early literacy t2	8.67	2.86	8.81	2.60	8.63	2.77	8.84	2.48	8.74	2.68

*Note*: For control variables and t1 assessments *N* = 497–500; for t2 assessments *N* = 491–495. Migration background: 0 = no migration background, 1 = one or both parents were born abroad; MARKO‐S = mathematical screening.

Abbreviation: SES, socioeconomic status.

Table S4 (available at https://osf.io/3ca6h/) shows the correlations for all study variables for the total sample. As expected, all literacy and numeracy subtests were significantly correlated with each other and with children's intelligence and the families' SES. Girls outperformed boys in some literacy subtests and boys were better than girls in some of the numeracy subtests. Children with migration background were outperformed by their peers without migration background in many subtests, in particular in measures of vocabulary. Finally, older children showed better outcomes in several subtests.

### Intervention effects (intention‐to‐treat)

**FIGURE 1:**
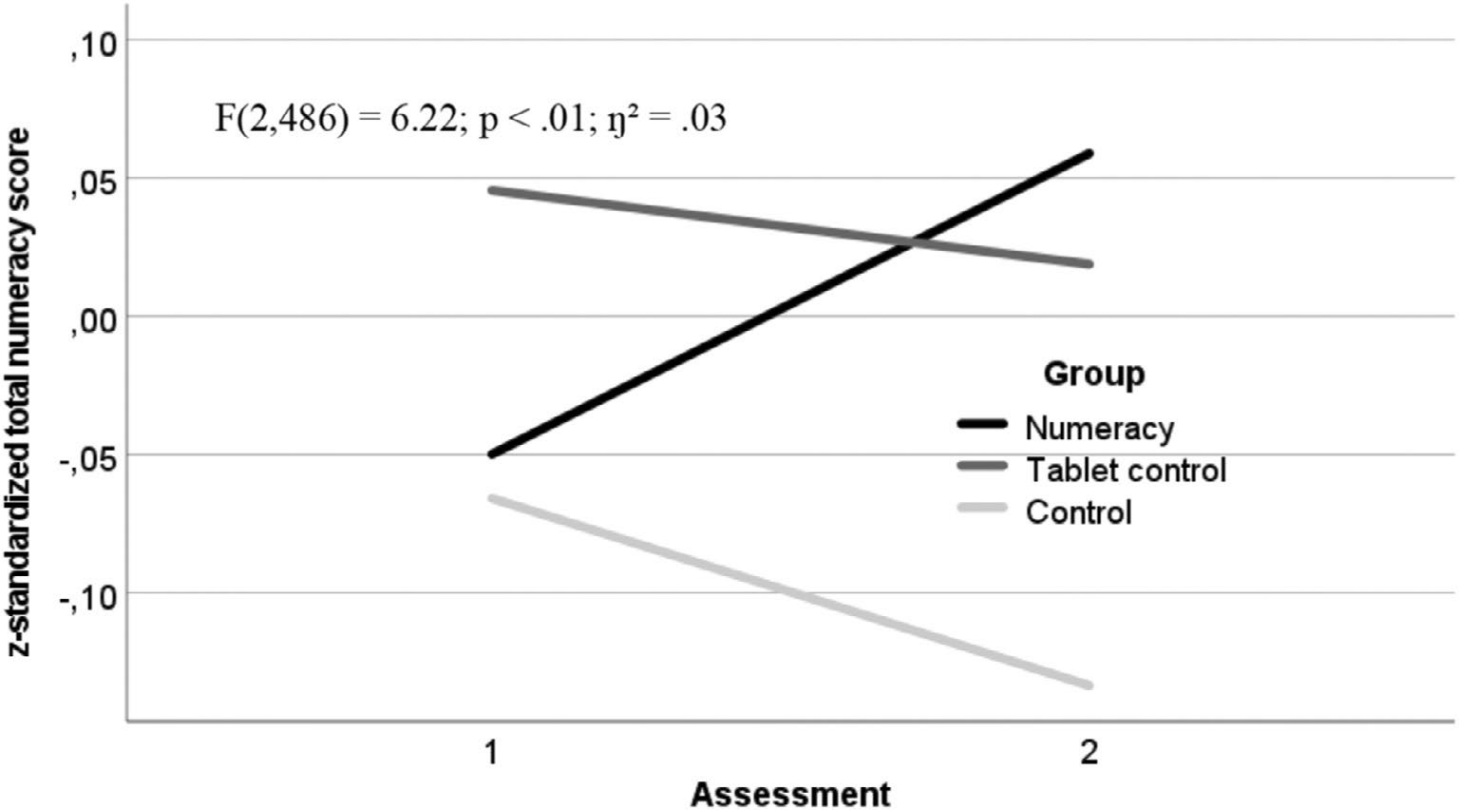
Change in the numeracy scores across the intervention period for the numeracy intervention, the tablet‐control, and the control groups. Standardized scores were analyzed and that a decline does not indicate lower numeracy scores at t2 compared to t1. Controlled for child age, sex, and intelligence and family migration background and socioeconomic status.

**FIGURE 2:**
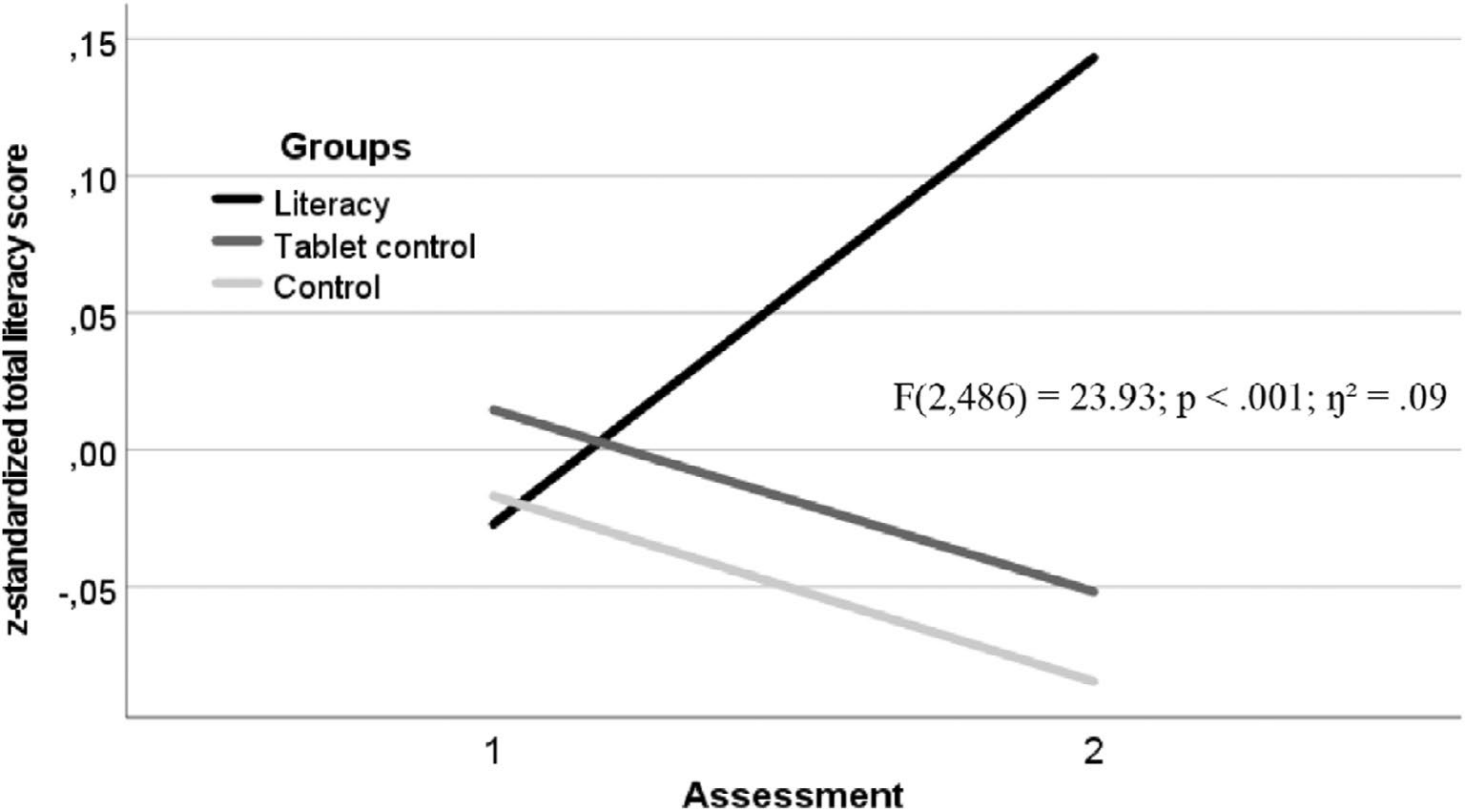
Change in the literacy scores across the intervention period for the literacy intervention, the tablet‐control, and the control groups. Standardized scores were analyzed and that a decline does not indicate lower literacy scores at t2 compared to t1. Controlled for child age, sex, and intelligence and family migration background and SES.

When controlled for child age, sex, intelligence, family migration background, and family SES, children in the numeracy group showed a greater gain in mathematical competencies (*F*(2, 486) = 6.22; *p* < .01; *η* ^2^ = .03, see Figure 1). An even greater effect was found for the literacy intervention, when controlling for the same child and family characteristics (*F*(2, 486) = 23.93; *p* < .001; *η* ^2^ = .09, see Figure 2). Consequently, the intervention approach was successful, in general, in supporting children's literacy and numeracy learning in the ecologically valid context of the family.

In a next analytic step, we conducted more detailed ITT analyses to test the robustness of the findings across cohorts and groups (see results in Appendix S1). When the intervention effects were tested for all four groups (i.e., literacy, numeracy, tablet‐control, control instead of a combined tablet‐control group), the results remained unchanged (see Figures S1 and S2). The same applied to separate analyses for both cohorts (see Figures S3–S6) with the exception of the intervention effect of the numeracy intervention in Cohort 1, which missed significance (*p* < .06). However, the effect size was somewhat larger than in the global test (*η* ^2^ = .03), so that this non‐significant result can be attributed to the smaller sample size (*n* = 187).

Next, we were interested in identifying specific literacy and mathematical competencies that have been trained by our tablet‐based intervention approach. Here, instead of the global numeracy and literacy total test scores, the same ITT analyses were conducted with specific subtests (see Table S1). Our numeracy intervention was particularly successful in enhancing children's number (symbol) knowledge and their backwards counting. The largest effect size for our literacy intervention was found for passive and active letter knowledge. In addition, the intervention supported children's gains in PA.

### Intervention effects (per protocol)

In addition to the ITT analyses, we also conducted per‐protocol analyses. Actual app usage times for each individual app and each child was assessed with mobile sensing and we were thus able to obtain ecologically valid data and consider intervention fidelity. In regression analyses with the total sample and controlled for initial competencies and the child and family control variables, a greater app usage time was associated with greater gains in mathematical competencies (see Table 2).

**TABLE 2:** Results of the regression analyses to predict numeracy outcomes at t2.

Model	Variables	*B*	SE	*β*	*T*	*p*
1	Numeracy t1	.87	.02	.86	36.39	.00*
2	Numeracy t1	.81	.03	.79	29.36	.00*
	Sex (1 = girls; 0 = boys)	−.07	.04	−.04	−1.76	.08
	Age	.01	.00	.06	2.45	.01*
	Intelligence	.01	.00	.10	3.84	.00*
	SES	.05	.03	.05	2.00	.05*
	Migration background	.02	.04	.01	0.56	.57
3	Numeracy t1	.81	.03	.80	3.43	.00*
	Sex (1 = girls; 0 = boys)	−.05	.04	−.03	−1.44	.15
	Age	.01	.00	.05	2.24	.03*
	Intelligence	.01	.00	.09	3.68	.00*
	SES	.06	.03	.06	2.25	.02*
	Migration background	.01	.04	.01	0.28	.78
	Total usage time numeracy apps	.00	.00	.12	5.33	.00*

*Note*: Three models are presented with initial mathematical competencies as predictor in model 1 (*R* ^2^ = .73), child and family control variables as additional predictors in model 2 (Δ*R* ^2^ = .02), and total usage time of numeracy apps as additional predictor in model 3 (Δ*R* ^2^ = .01). Migration background: 0 = no migration background, 1 = one or both parents were born abroad. Significant results are marked with an asterisk (*).

Abbreviation: SES, socioeconomic status.

In our study, a playing time of about 750 min in total, which is about 4.5 min daily during our intervention phase resulted in a gain of 0.1 SD in mathematical competencies. Similarly, a literacy app usage time of about 430 min, which is about 2.5 min daily during the intervention period led to a gain of 0.1 SD in literacy competencies on average (see Table 3). Consequently, our findings show that children's gains in literacy and mathematical competencies were associated with the actual time spent playing the learning apps. Results remained unchanged, even when three outliers with more than 5500 min of total math app usage were excluded from the analyses.

**TABLE 3:** Results of the regression analyses to predict literacy outcomes at t2.

Model	Variables	*B*	SE	*β*	*T*	*p*
1	Literacy t1	0.87	0.02	.86	36.79	.00*
2	Literacy t1	0.75	0.03	.74	26.15	.00*
	Sex (1 = girls; 0 = boys)	0.02	0.03	.02	0.73	.47
	Age	0.01	0.00	.06	2.52	.01*
	Intelligence	0.01	0.00	.11	4.30	.00*
	SES	0.07	0.02	.09	3.24	.00*
	Migration background	−0.09	0.04	−.07	−2.60	.01*
3	Literacy t1	0.75	0.03	.74	27.26	.00*
	Sex (1 = girls; 0 = boys)	0.03	0.03	.02	1.03	.30
	Age	0.01	0.00	.06	2.65	.01*
	Intelligence	0.01	0.00	.11	4.77	.00*
	SES	0.09	0.02	.09	3.73	.00*
	Migration background	−0.09	0.03	−.07	−2.83	.01*
	Total usage time literacy apps	0.00	0.00	.15	6.93	.00*

*Note*: Three models are presented with initial literacy competencies as predictor in model 1 (*R* ^2^ = .74), child and family control variables as additional predictors in model 2 (*ΔR* ^2^ = .02), and total usage time of literacy apps as additional predictor in model 3 (*ΔR* ^2^ = .02). Migration background: 0 = no migration background, 1 = one or both parents were born abroad. Significant results are marked with an asterisk (*).

Abbreviation: SES, socioeconomic status.

In a last analytic step, we tried to predict specific literacy and numeracy outcomes with the total usage time of specific learning apps that trained these outcomes (see Table S2). Indeed, the total usage time of apps that focused on letter knowledge was a significant predictor of the change in passive and active letter knowledge when initial letter knowledge and child and family characteristics were controlled for. Furthermore, the total usage time of apps that focused on PA was a significant predictor of the change in rhyming competencies and initial sound identification between t1 and t2. Similarly, children's gain in number knowledge was predicted by the total usage time of apps focusing on numbers and number symbols and the change in children's counting forward, backwards and identifying precursor and successor numbers was predicted by math apps in which counting was the focus.

## DISCUSSION

We demonstrate that a digital, app‐based family intervention with newly developed learning apps that were designed according to educational and psychological theories (Hirsh‐Pasek et al., 2015) was able to improve children's development of both literacy and mathematical competencies and thus facilitate children's cognitive school readiness. Furthermore, our findings show that a longer usage of specific apps was associated with greater gains in the specific competencies they train. Consequently, the results provide support for all our hypotheses. Given the increasing prominence of tablet‐style portable devices and internet access worldwide (Chaudron et al., 2018) such interventions have the potential to support all children in their competencies' development as a low‐cost, high‐benefit method. Altogether, our findings point out that the app‐based intervention was successful in an experimental control‐group design that controlled for important child and family characteristics.

Such a digital tablet‐based intervention with appropriate learning apps can be applied successfully in the context of family learning and train important basic literacy and mathematical competencies and thus precursors of later academic abilities (Niklas & Schneider, 2017a; Stock et al., 2009; Torppa et al., 2007). This outcome is even more meaningful, when the multibillion‐dollar educational app market with hundreds of millions of downloads is considered (Statista, 2021). Despite countless learning apps being available for mobile devices, only a minority of them has ever been evaluated. Furthermore, even learning apps used in scientific studies may be detrimental to children's competencies development (Kim et al., 2021). Altogether, long screen times can have a negative influence on children's sleeping quality (Janssen et al., 2020) and behavior (Eirich et al., 2022).

In contrast, the learning apps used in our intervention had several specific features that may have contributed to their effectiveness for children's learning (Hirsh‐Pasek et al., 2015). First, they were explicitly developed without distracting elements such as popping up animations, distracting sounds, and background music. Second, they all promoted active cognitive learning with a level structure and increasing difficulty, so that all children were able to profit from them independent of their competency level and background. Third, all apps in our intervention groups had a specific learning goal and aimed to improve key literacy and mathematical competencies.

In our study, we have shown that using appropriate learning apps in the family has the potential to support children's learning independent of their SES and at a critical developmental time in life (Heckman, 2006). Consequently, our low‐cost intervention has the potential to prepare children for school by enhancing important literacy and mathematical competencies and thus to also enhance children's academic success in the long run. Similar to previous studies (e.g. Berkowitz et al., 2015), our findings indicate that longer app‐usage times were associated with greater competencies' gains.

Our results imply that it is not necessary for children to play the learning apps for very long time to achieve meaningful gains in their competencies. Instead, a few minutes of regular daily game play seems to be sufficient which also aligns with recommendations of limited screen time for young children (Eirich et al., 2022; Fang et al., 2019; Janssen et al., 2020). Future studies should test whether the total usage time is decisive, whether there is a maximum usage time beyond which no more meaningful gains are found, and whether the frequency of app usage matters (e.g., short daily app usage vs. longer app usage once a week).

Our analyses showed that the numeracy intervention was particularly successful in enhancing children's number (symbol) knowledge and their backwards counting. Skills like these have been identified as important precursors of mathematical competencies later in school (Niklas & Schneider, 2017a; Stock et al., 2009). The largest effect sizes in our analyses were found for passive and active letter knowledge. In addition, the intervention supported children's gains in PA. Both PA and letter knowledge are considered important precursors of later reading and writing abilities in school (Niklas & Schneider, 2013, 2017a; Torppa et al., 2007).

More specifically, our learning apps were also successful in training exactly the competencies that were targeted during the usage of the apps. For instance, our apps with a focus on number symbol knowledge were successful in training exactly this knowledge and the same applied to the apps focusing on either counting, letter knowledge, or PA. Here, it would be of interest whether these gains are sustained for a longer period of time and whether children in the intervention groups indeed are better prepared when entering schools.

### Implications

The findings of our study indicate that high‐quality learning apps are able to support children's learning. However, we also know that there are numerous low‐quality apps on the market (Meyer et al., 2021) for which no such support of children's competencies can be expected; or even worse, which may even have a detrimental effect (see Kim et al., 2021). Consequently, app developers should work together closely with educational experts and should consider important quality aspects of apps (Hirsh‐Pasek et al., 2015), when programming learning apps for children.

Furthermore, it becomes clear that practitioners and parents are in need of guidance. It is in their interest to assist and enhance young children's learning and many of them already use digital media to support children's learning processes. However, most of them are not experts in the evaluation of learning apps and may chose low‐quality apps, despite best intentions. It would be helpful, if more transparent, current, objective, and readily understandable app evaluations would be freely available as guidance for the public.

The usage and evaluation of learning apps is a fairly recent field for research and more studies, in particular, on young children's learning with digital media is needed (Chaudron et al., 2018). Research should focus on more experimental field studies that test the usage of high‐quality learning apps in different contexts such as kindergartens and families. Furthermore, we are only beginning to understand the learning mechanisms that can be triggered by such apps (Hirsh‐Pasek et al., 2015) and studies should analyze in which way learning happens, when children interact with digital devices. Finally, we still do not know, whether any learning gains from the usage of learning apps are sustained in the long term. Therefore, more longitudinal research with follow‐up‐assessments is needed.

### Limitations

Some study limitations should be noted. Despite our efforts, the average SES in our sample was medium to high and only a few families with a low SES participated. However, we controlled for SES in our analyses and in an exploratory check with families with a low SES only, the same results were found.

Furthermore, due to technical problems, the usage times of some families were missing and some families did not receive all apps as intended (i.e., late and/or for a shorter period of time as intended). Moreover, we only assessed the app usage times; however, we cannot be certain whether and how often the study child used the apps alone or together with other family members or friends. As our study is a field experiment such limitations were to be expected and they add to the ecological validity of our study results.

In addition, only immediate intervention effects are analyzed, and we therefore cannot be sure whether these effects are to be found at follow‐up assessments. However, given the high stability of cognitive child competencies (e.g., Krajewski & Schneider, 2009), some longer‐term advantages for the children in the intervention group are to be expected.

Furthermore, EFs that play a critical role in children's early learning were not the main focus of our study and thus were not assessed. However, we acknowledge that in the learning processes triggered by some of the used learning apps, EFs will have played an important role (e.g., working memory, inhibition, planning). In particular, the learning apps provided to the tablet‐control group may be associated with children's EFs.

### Strengths

Despite these limitations several strengths mark the present study. We were able to assess two large samples two times to test for potential intervention effects. Furthermore, a comprehensive test battery was used to assess various important early literacy and mathematical competencies in a standardized test procedure. In our ecologically valid approach (e.g., applying mobile sensing in the family context), we provided the tablets with our learning apps for home usage in the family context without further obligations for the families. Consequently, usage times differed for children according to their individual interest and daily schedules. Here, we were able to show that longer usage times led to greater competencies gains.

In addition, we did not focus on either literacy or mathematical competencies, but included both competencies in our intervention design. Furthermore, we included not only a control group without tablets in our study design but also a second control group, in which children also received the same tablets for home usage. In this group, children were provided with other learning apps that did not focus on literacy and numeracy. Even when compared with such an active control group, our intervention groups showed significantly greater gains in their competencies' development.

## CONCLUSION

Our findings show that an app‐based family intervention study can support children's competencies development in the year before school entry. Given the importance of digital media even in young children's daily life, high‐quality learning apps may play an important role in preparing children for a successful school entry. As children have access to digital media independent of their families' SES and migration background, app‐based interventions may also be able to support these children who are most in need of such additional support.

## AUTHOR CONTRIBUTIONS

FN is the PI of this study and wrote the first draft of the manuscript. EB, AM, and AW were responsible for the acquisition of the data. EB, AM, and AW carried out part of the assessments. FN did the analysis. All the authors contributed to the manuscript and read and approved the submitted version.

## FUNDING INFORMATION

This project has received funding from the European Research Council (ERC) under the European Union's Horizon 2020 research and innovation program (Grant agreement No 801980).

## ETHICS STATEMENT

This study was performed in line with the principles of the Declaration of Helsinki. Approval was granted by the ethics committee of the Faculty of Psychology and Educational Sciences, University of Munich (LMU).

## Supporting information


Appendix S1.

## Data Availability

All data and material are available from FN, upon reasonable request. The data necessary to reproduce the analyses presented here are not publicly accessible yet, but will be made available after the project is completed. The analytic code necessary to reproduce the analyses presented in this paper is not publicly accessible. The materials necessary to attempt to replicate the findings presented here are not publicly accessible. The analyses presented here were not preregistered, however, the general analytic approach that includes the analyses presented here, was described in the Grant agreement before the start of the project.
