# Supplementary material for: Learning apps at home prepare children for school
Source: Child Dev. 2024 Oct 24;96(2):577–90. doi: 10.1111/cdev.14184 (PMC11868692; doi:10.1111/cdev.14184)
Supplement: Supplementary file 1 — Appendix S1. [file CDEV-96-577-s001.zip › cdev14184-sup-0001-Supinfo.docx]

Supplementary Materials for

Quality learning apps at home prepare children for school

**This file includes:**

Tables S1 to S3

Figs. S1 to S6

Correlation table S4 (not included; available at <https://osf.io/3ca6h/>)

**Table S1**

*Results for the Repeated Measurement ANCOVAs to Predict Change in Subtest Outcomes across t1 and t2 with Group as Factor.*

| **Outcome variable** | ***F*** | ***p*** | ***ŋ²*** |
| --- | --- | --- | --- |
| MARKO-S | 1.78 | .17 | .01 |
| **Counting backwards** | **3.96** | **.02** | **.02** |
| Counting forwards | .51 | .60 | .00 |
| Predecessors and successors | 1.69 | .19 | .01 |
| **Number (symbol) knowledge** | **9.23** | **< .001** | **.04** |
| Calculating | .16 | .85 | .00 |
| Grammar | .17 | .84 | .00 |
| Rhyming task | 2.45 | .09 | .01 |
| **Letter sound identification** | **7.49** | **< .001** | **.03** |
| Active vocabulary | 2.69 | .07 | .01 |
| Passive vocabulary | .05 | .95 | .00 |
| **Active letter knowledge** | **41.47** | **< .001** | **.15** |
| **Passive letter knowledge** | **24.46** | **< .001** | **.09** |
| Early literacy | .39 | .68 | .00 |

*Note.* Results for the numeracy intervention group are presented for MARKO-S until Calculation and results for the literacy intervention group are presented for Grammar until Early literacy. Significant results are in bold print. Controlled for child age, sex, and intelligence and family migration background and socio-economic status.

Table S2

*Results for the Regression Analyses to Predict Specific Outcomes by Specific App Usage Times.*

| **Variables** | ***B*** | ***SE*** | ***β*** | ***T*** | ***p*** | ***ΔR²*** |
| --- | --- | --- | --- | --- | --- | --- |
| Number knowledge | .00 | .00 | .13 | 4.46 | <.001 | .02 |
| Counting backwards | .00 | .00 | .16 | 4.75 | <.001 | .03 |
| Counting forwards | .00 | .00 | .08 | 2.47 | .01 | .01 |
| Predecessors and successors | .00 | .00 | .10 | 2.98 | <.01 | .01 |
| Rhyming task | .00 | .00 | .09 | 3.84 | <.01 | .01 |
| Letter sound identification | .00 | .00 | .19 | 4.98 | <.001 | .04 |
| Active letter knowledge | .00 | .00 | .26 | 9.49 | <.001 | .07 |
| Passive letter knowledge | .00 | .00 | .24 | 7.19 | <.001 | .06 |

*Note.* All regression analyses are controlled for the outcome at t1, child age, sex, and intelligence, family migration background, and socioeconomic status of the family.

Table S3

*Overview of the Learning Apps Used in the [blinded] Study*

| **App Name** | **Month** | **Description – Literacy Apps** | **Screenshot** | **Trained Skills** |
| --- | --- | --- | --- | --- |
| Memory (Letters) | 1 | This app corresponds with the traditional analogue memory game. All cards exist in pairs and are placed face down. Children turn the cards over by tapping and memorize the letters on them. The aim of the game is to uncover two matching cards directly after each other (e.g., “A” and “A” or “A” and an image of an “Ape”). Letter sounds are provided when tapping. | 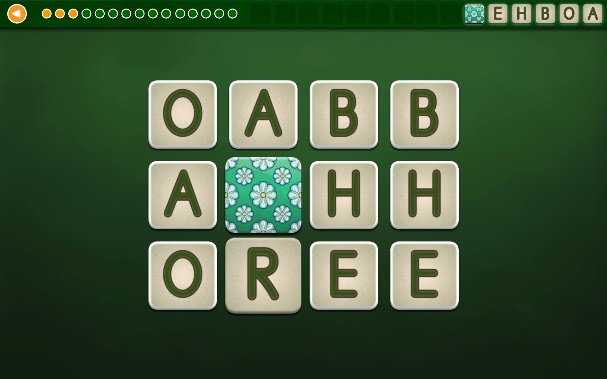 | Active and passive letter knowledge; phonological awareness |
| Letter drawing | 1 | In this app, letters are to be traced with the finger following displayed lines. Children follow a displayed arrow that guides them tracing letters. Letter sounds are provided for each item. | 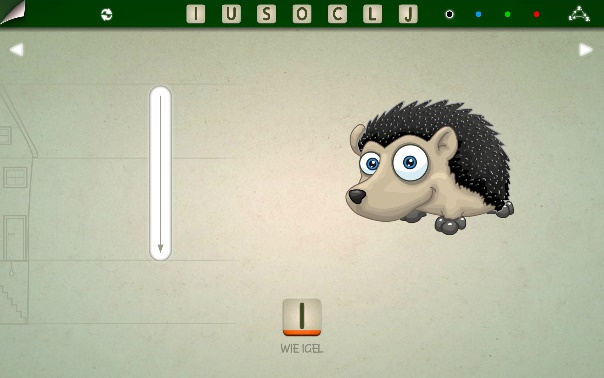 | Active and passive letter knowledge |
| Painting with letters | 1 | In this app, children colour pictures. Both the areas of the picture and the colours are marked with letters. E.g., an area marked with “A” is to be coloured with the colour marked with “A” (e.g., blue). Letter sounds are provided when tapping. | 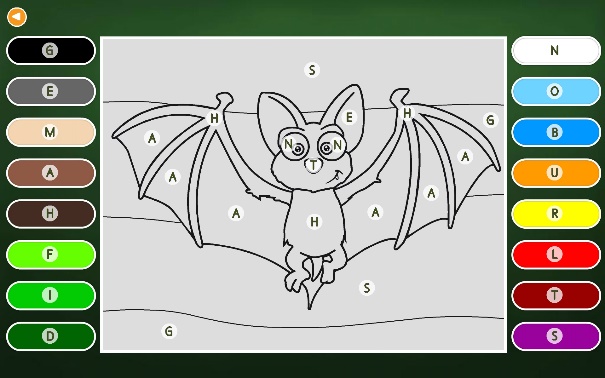 | Active and passive letter knowledge |

| **App Name** | **Month** | **Description – Literacy Apps** | **Screenshot** | **Trained Skills** |
| --- | --- | --- | --- | --- |
| Letter sorting | 1 | In this app, children sort a series of letters (e.g., D-A-B-C) in the correct order according to the alphabet (e.g., A-B-C-D). Pictures of animals with the corresponding initial letter simplify the matching. Letter sounds are provided for each item. | 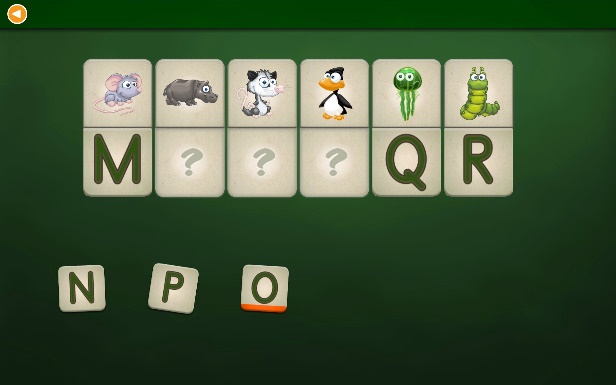 | Active and passive letter knowledge, phonological awareness |
| Initial letter sounds | 2 | In this app, individual initial sounds of words have to be identified. In each level a target initial sound (e.g. “L”) has to be differentiated from other initial sounds (e.g., “F”). Children are presented with animals one after the other (e.g., cow, lynx, lion, whale). Animals with the same initial sound as the target sound need to be selected (e.g., lynx, lion). | 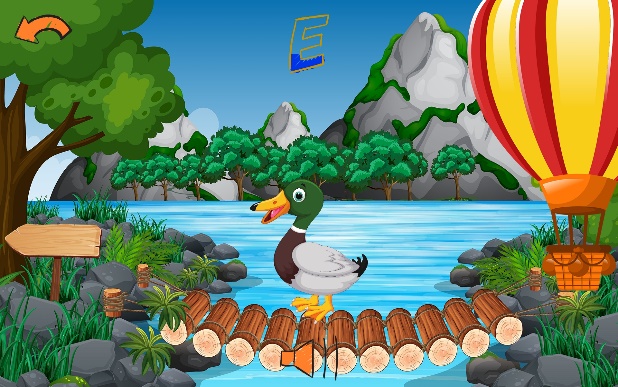 | Phonological awareness |
| Snakes & Ladders (Letters) | 2 | This app corresponds to the traditional Snakes & Ladders game. The board and the dice are marked with letters. This game can be played in multi-player mode. Letter sounds are provided. | 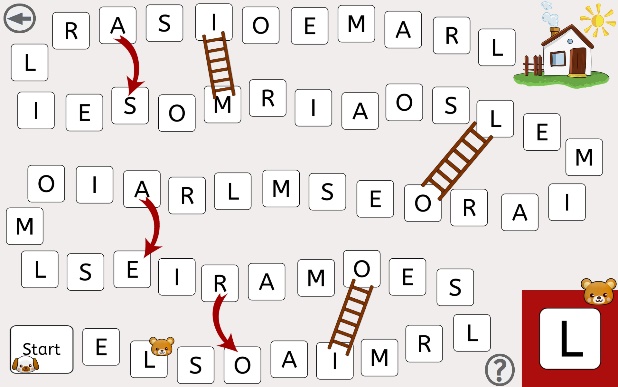 | Active and passive letter knowledge |

| **App Name** | **Month** | **Description – Literacy Apps** | **Screenshot** | **Trained Skills** |
| --- | --- | --- | --- | --- |
| Find the vowels | 3 | In this app, a letter is presented visually and audibly to the children (e.g., “A”). The children’s task is to select the correct object/animal out of two, three or four presented objects/animals that incorporates the target letter/sound (e.g., “panda”). | 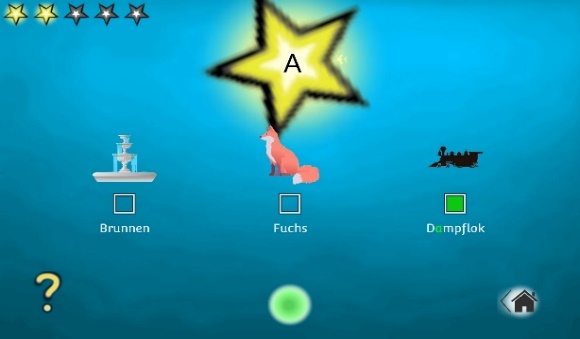 | Phonological awareness |
| Finding pairs (rhymes) | 3 | In this app, the image of an object (e.g., “Wind”) is visually and audibly presented to the children. They must now select one out of four images, which rhyme(s) with the presented object (e.g., “Kind”). Each word is provided to the child when tapping on it. | 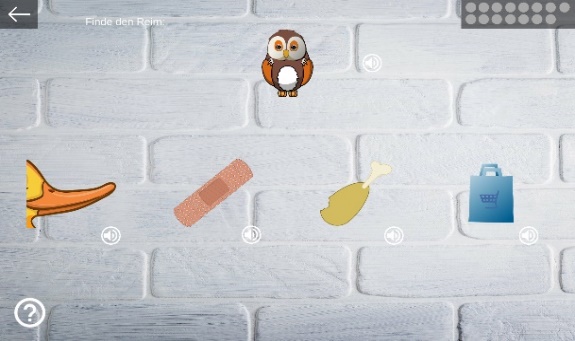 | Phonological awareness |
| Sentence understanding | 4 | In this app, children are read a short story. After each paragraph, they are asked several questions about the content of the story, which they can answer by selecting the correct image out of four using images depicting the answer options (e.g., “Do you remember what season it was?”). | 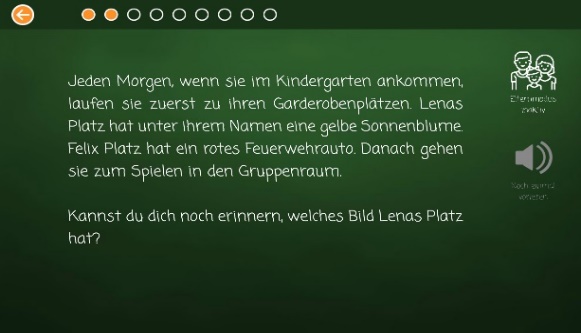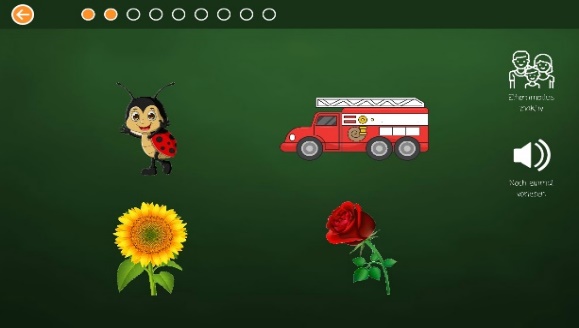 | Passive vocabulary; early literacy knowledge |

| **App Name** | **Month** | **Description – Literacy Apps** | **Screenshot** | **Trained Skills** |
| --- | --- | --- | --- | --- |
| Letter-Domino | 4 | In this app, the task is to place game tokens together like in a traditional analogue domino game. The game pieces have letters and/or animal faces on them. The same letters or animals with their initial sound needs to be matched. Animal names and letter sounds are provided verbally. | 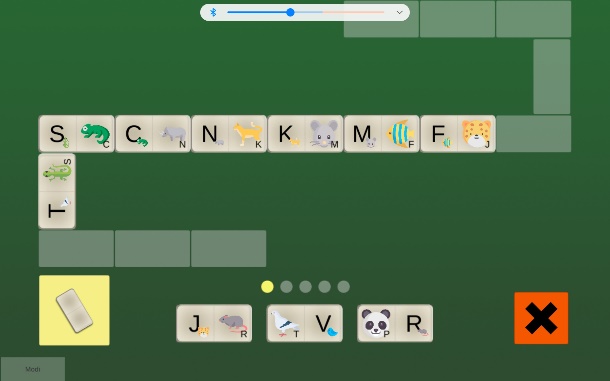 | Active and passive letter knowledge, phonological awareness |
| Word-Puzzle^1^ | 5 | In this app, children see a target word with an image of the target. Their task is to connect the correct letters of this word in a letter cloud below the item. | 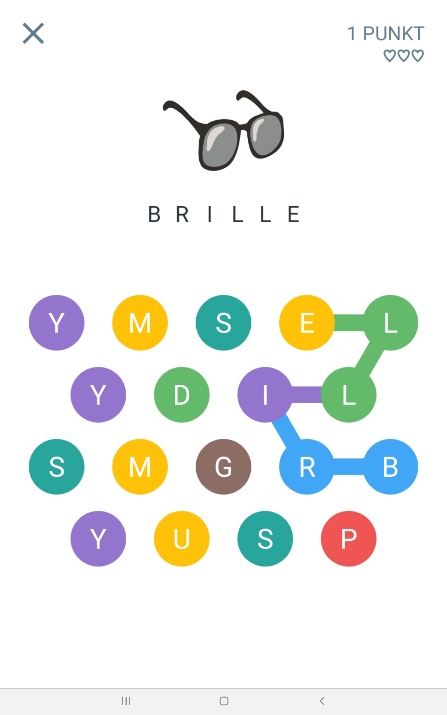 | Letter knowledge, vocabulary, early literacy knowledge |
| Magic potion (sounds) | 5 | In this app, children help a witch to brew magic potions, for which they need various ingredients. The witch spells out the sounds of the object that is needed next (e.g., P-O-T). The children must identify the correct ingredient (e.g., the pot) and select it from an array of distractors. | 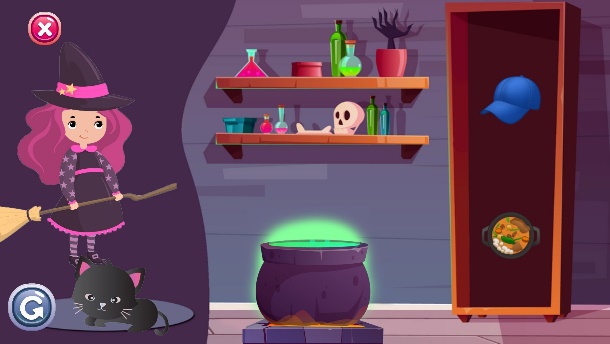 | Phonological awareness |

| **App Name** | **Month** | **Description – Numeracy Apps** | **Screenshot** | **Trained Skills** |
| --- | --- | --- | --- | --- |
| Memory (Numbers) | 1 | This app corresponds with the traditional analogue memory game. All cards exist in pairs and are placed face down. Children turn the cards over by tapping and memorize the numbers on them. The aim of the game is to uncover two matching cards directly after each other. Number names are provided verbally when tapping in the easier levels. | 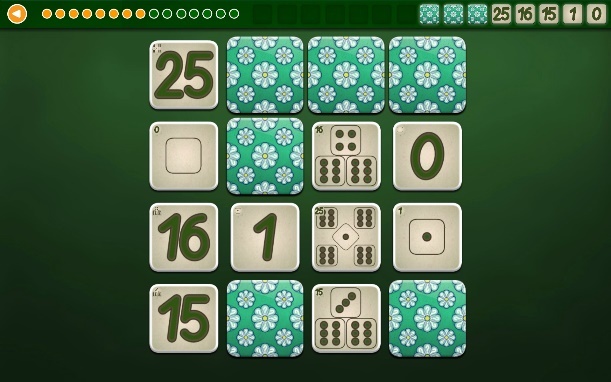 | Number symbol knowledge, knowledge of numeric representation |
| Number drawing | 1 | In this app, the aim is to trace the lines with your finger following displayed lines. Children follow a displayed arrow and then trace whole number symbols. Number names are provided verbally for each item. | 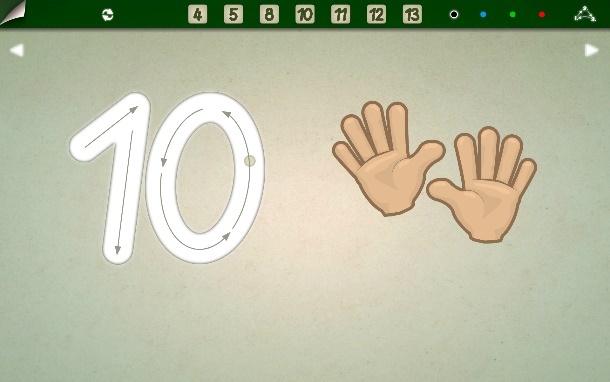 | Number symbol knowledge, knowledge of numeric representation |
| Painting with numbers | 1 | In this app, children colour pictures. Both the areas of the picture and the colours are marked with numbers. The colours to be used for certain areas are linked and displayed using a legend with numbers. Number names are provided verbally when tapping. | 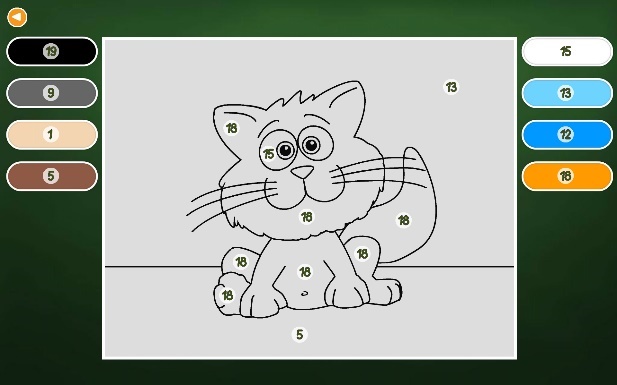 | Number symbol knowledge |

| **App Name** | | **Month** | | **Description – Numeracy Apps** | | | **Screenshot** | **Trained Skills** | |
| --- | --- | --- | --- | --- | --- | --- | --- | --- | --- |
| Number sorting | 1 | | In this app, children sort a series of numbers in the correct order. The children's task is to correctly assign the numbers, which are offered below in a randomized order and in different forms of representation. Number names are provided verbally. | | | 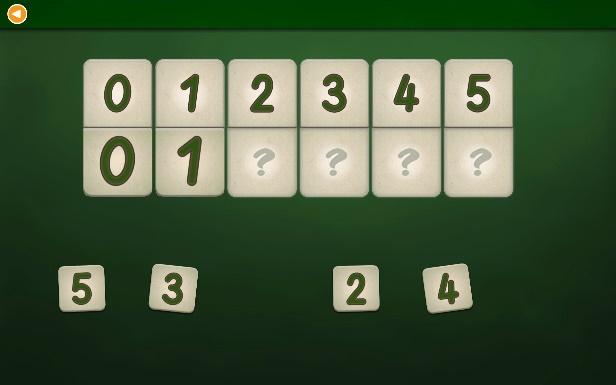 | | | Number symbol knowledge, Ordinal number knowledge, |
| Build a number rocket | 1 | | In this app, children build a number rocket. On the right-hand side they see a rocket with empty fields and on the left side a selection of rocket components with different numbers. The rocket must be constructed with equal/ascending/descending number sequences. Number names are provided verbally. | | | 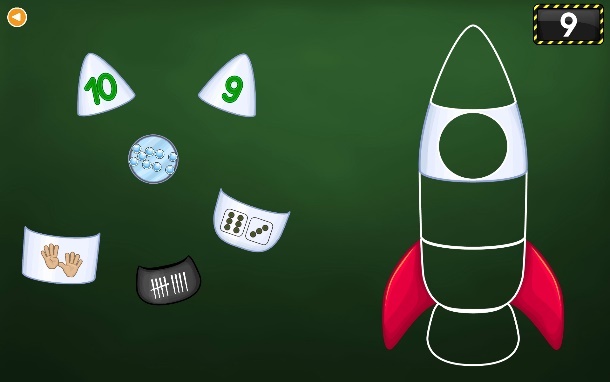 | | | Number symbol knowledge, knowledge of numeric representations, ordinal number knowledge |
| Collecting nuts (numbers) | 2 | | In this app, a squirrel runs along a predetermined path to a destination. The children are provided with the information how many (different) nuts the squirrel needs and they are asked to tap the number of nuts accordingly. | | 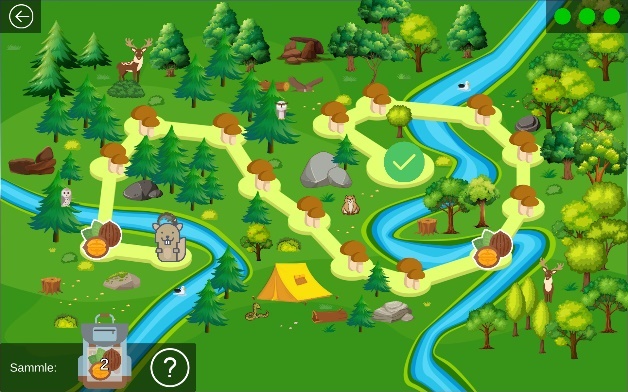 | | | Counting, inclusion and relations, number sequence forwards, cardinal number knowledge | |

| **App Name** | | **Month** | | **Description – Numeracy Apps** | | **Screenshot** | **Trained Skills** | |
| --- | --- | --- | --- | --- | --- | --- | --- | --- |
| Snakes & Ladders (Numbers) | 2 | | This app corresponds to the traditional Snakes & Ladders game. The board and the dice are marked with numbers. This game can be played in multi-player mode. Number names are provided verbally. | | 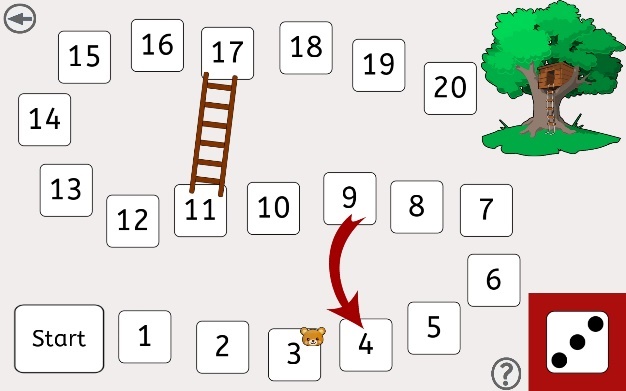 | | | Number symbol knowledge, number sequence forwards, number sequence backwards, number predecessors and successors |
| Tap it! Numbers | 2 | | In this app, children should select numbers by tapping them as quickly as possible. The children are instructed to find and tap the target number “x”. The children are asked to tap the indicated number “x” and not the 1-3 distractor numbers that also appear regularly. After tapping (or when the numbers were not tapped on fast enough), the numbers disappear. In a second game phase, children are asked to count. | | 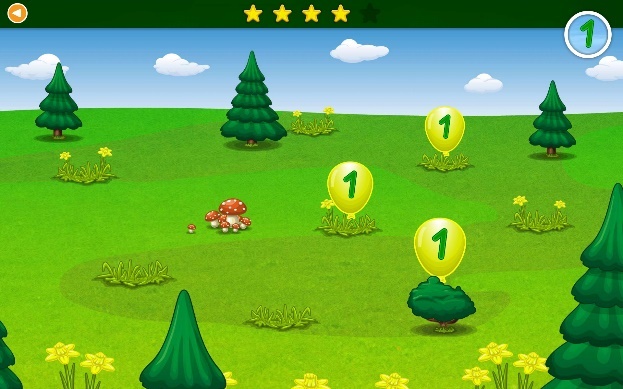 | | | Number symbol knowledge; knowledge of numeric representations,  number sequence forwards, number sequence backwards |
| Mathemarmite^1^ | 2 | | In this app, children create monsters by using their counting abilities to put the correct number of specific ingredients into a magic pot. The game mainly contains counting tasks, but different representations for the numbers. | | 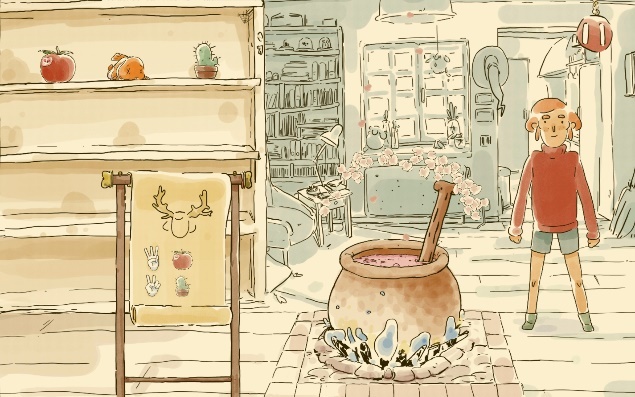 | | | Number symbol knowledge, counting, knowledge of numeric representations |

| **App Name** | | **Month** | | **Description – Numeracy Apps** | | **Screenshot** | **Trained Skills** | |
| --- | --- | --- | --- | --- | --- | --- | --- | --- |
| Measurement app | 3 | | In this app, children see a ruler in the middle of the screen. Objects and animals of different sizes appear next to the ruler. There is a number box under the ruler and the children should measure the correct size and choose the corresponding number card. In a later level, they also should build the size of the objects/animals with building blocks. | | 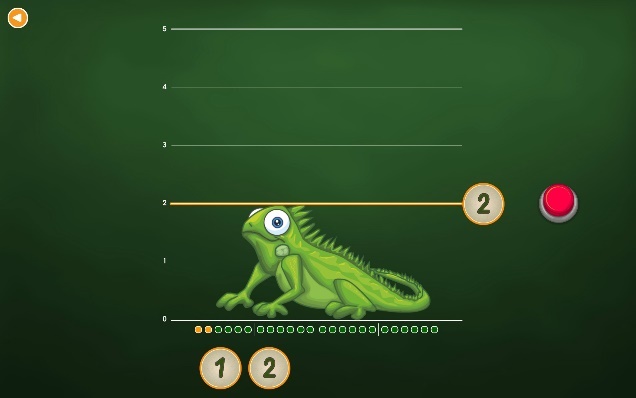 | | | Number symbol knowledge, inclusion and relations, comparison and measurement, easy calculations |
| Finding pairs (Numbers) | 3 | | In this app, children are asked to choose the appropriate object out of two to four objects, that corresponds to a given number. The numbers are provided verbally. | | 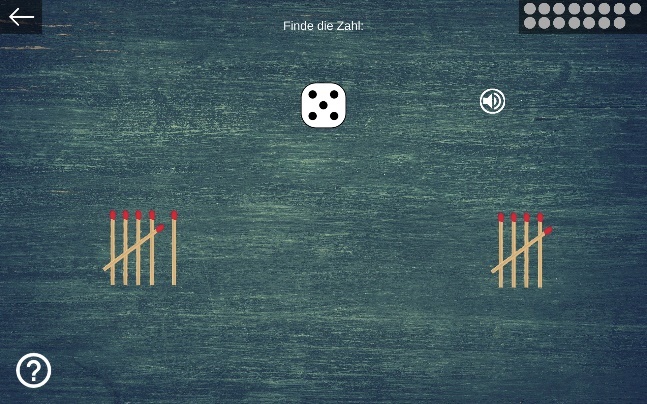 | | | Number symbol knowledge; Knowledge of numeric representations |
| Count and compare | 3 | | In this app, children have to count the number of animals correctly and choose the correct number card. Later, they have two identify the correct number of animals according to a given number symbol that is also named verbally. In the final levels, the quantities need to be compared (larger, smaller, equal). | | 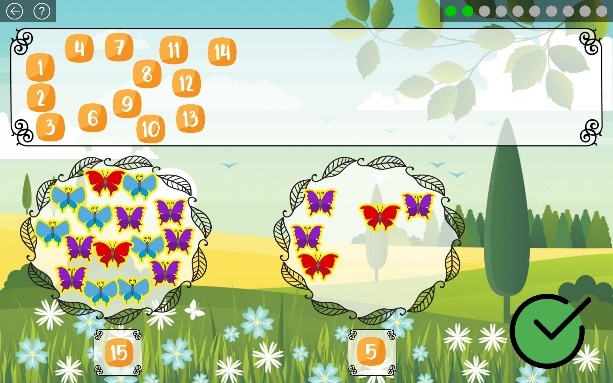 | | | Number symbol knowledge, ordinal number bars, cardinality, inclusion and relations, counting, number sequence forwards, number sequence backwards, |

| **App Name** | | **Month** | | **Description – Numeracy Apps** | | **Screenshot** | **Trained Skills** |
| --- | --- | --- | --- | --- | --- | --- | --- |
| Counting the balloons | 4 | | In this app, children are asked to count balloons and select the correct number of balloons by tapping. in later and more difficult levels, children are asked to do easy calculations with the balloons. | | 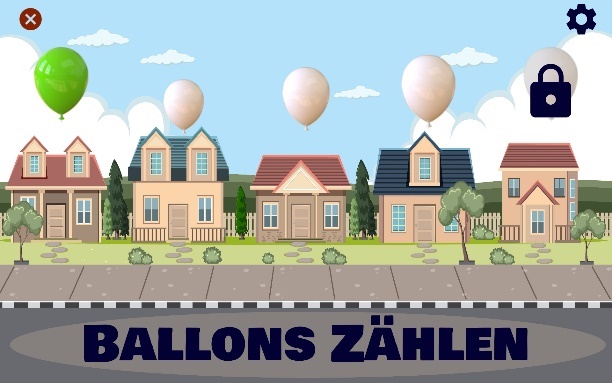 | | Number symbol knowledge, counting, number sequence forwards, number sequence backwards, addition & subtraction |
| Number-Domino | 4 | | In this app, children need to place the game pieces together like in a classic analogue domino game and solve the domino game correctly. The numbers on the domino tokens differ in size and representation. | | 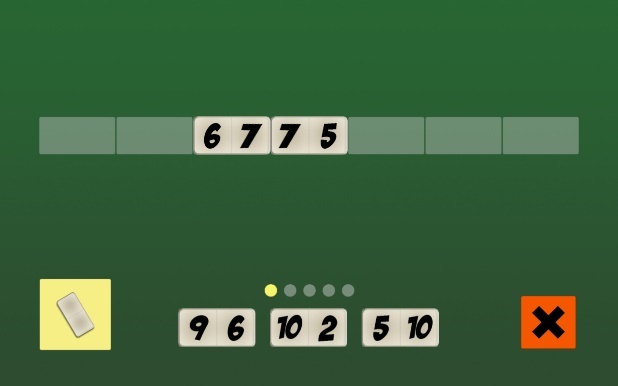 | | Number symbol knowledge, knowledge of numeric representations |
| Connect the number dots | 4 | | In this app, the task is to paint or complete a picture by connecting dots to form lines. The points to be connected are displayed with numbers. In later levels, the numbers go up to 20 and should be connected backwards. | | 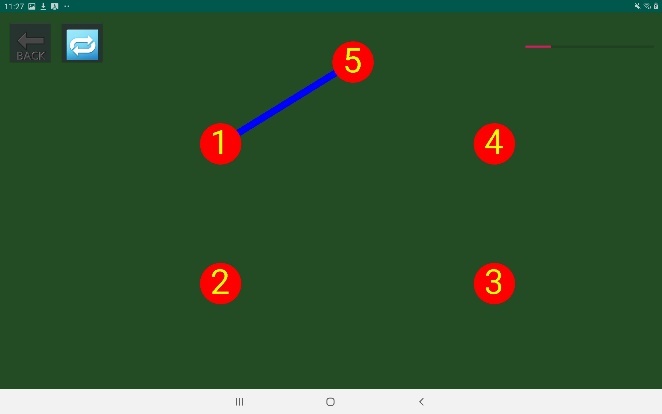 | | Number symbol knowledge, knowledge of numeric representations, counting, number sequence forwards, number sequence backwards |

| **App Name** | | **Month** | | **Description – Numeracy Apps** | | **Screenshot** | **Trained Skills** |
| --- | --- | --- | --- | --- | --- | --- | --- |
| Learn the Clock | 5 | | With this app, children learn the clock. First, times are displayed with whole hours. Later, children have to identify the time on the clock with smaller units. Finally, they have to conduct easy calculations with time periods. | | 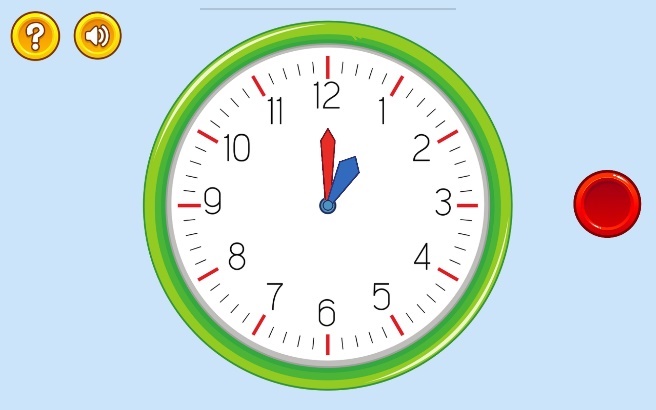 | | Number symbol knowledge, reading the clock, calculation with time. |
| Count and sort objects | 5 | | In this app, children have to count and assign different objects and number symbols to the corresponding number symbol on a box. | | 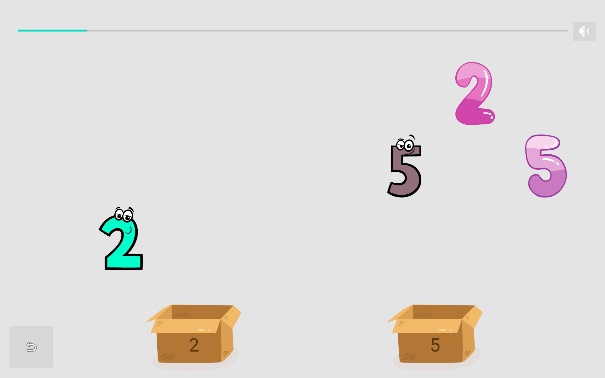 | | Number symbol knowledge, knowledge of numeric representations, |
| Categorize numbers | 5 | | In this app, children are supposed to find the identical objects and put them together in a row. Next, they have to count them and assign the correct number. | | 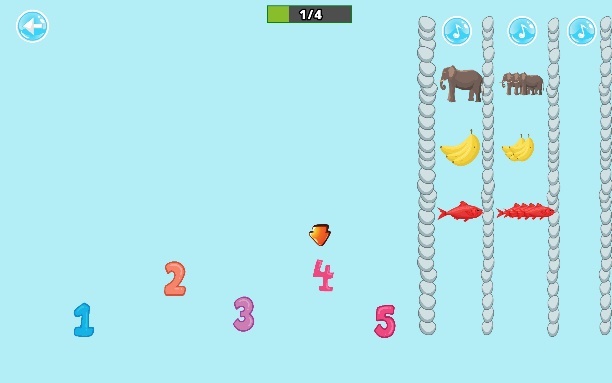 | | Number symbol knowledge, inclusion, counting |

| **App Name** | | **Month** | | **Description – Control Apps** | | **Screenshot** | **Trained Skills** |
| --- | --- | --- | --- | --- | --- | --- | --- |
| Memory (Colours) | 1 | | This game corresponds with the traditional analogue memory game. All cards exist in pairs and are placed face down. Children turn the cards over by tapping and memorize the colours on them. The aim of the game is to uncover two matching cards directly after each other (e.g., “purple” and “purple”). | | 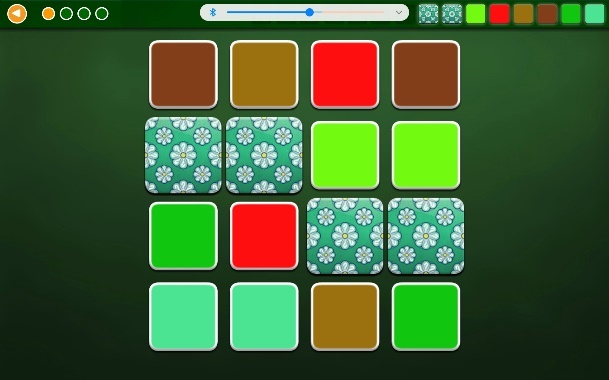 | | Short-term memory (STM), Working memory (WM), colour recognition |
| Line drawing (Labyrinth) | 1 | | In this app, children are expected to draw a line within given shapes (e.g., square, triangle) by using their fingertips. Children follow a displayed arrow that helps them to trace the line and complete the shapes. | | 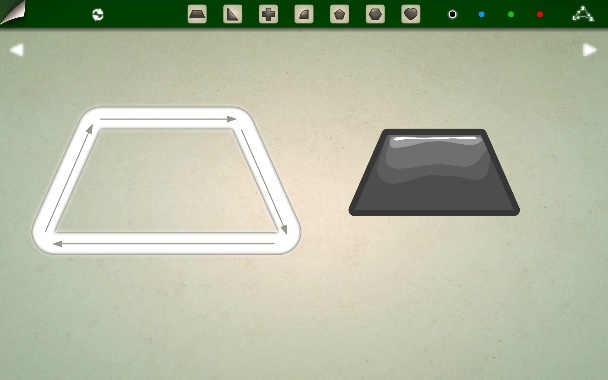 | | Hand-eye coordination |
| Painting with colours | 1 | | In this app, children select an empty drawing of their choice and paint this drawing by using shapes provided in both sides of the screen. | | 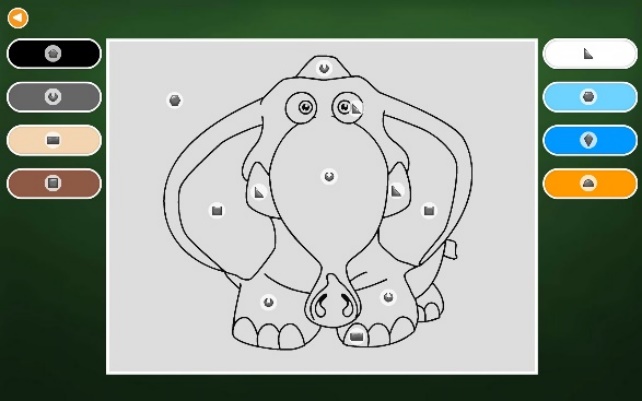 | | Hand-eye coordination, creativity |

| **App Name** | | **Month** | | **Description – Control Apps** | | **Screenshot** | **Trained Skills** |
| --- | --- | --- | --- | --- | --- | --- | --- |
| Animal puzzle | 1 | | This is a puzzle app, children are asked to put the puzzle pieces of animals together into a given animal’s shape (e.g., a frame in fish shape was given and the pieces of the puzzle were provided on the sides. After all the pieces are put together, a fish picture is visible) | | 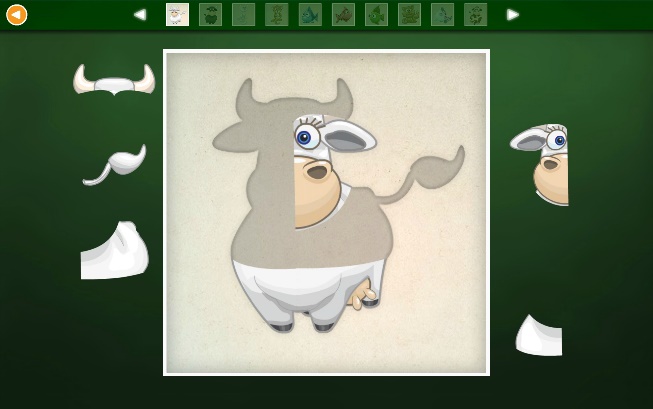 | | Problem solving, shape recognition, spatial sense |
| Build a colour rocket | 2 | | In this app, children build a rocket by assembling the parts of the rocket and inhibiting the given distractors. Each rocket part represents a different colour/shade of the same colour depending on the played level. | | 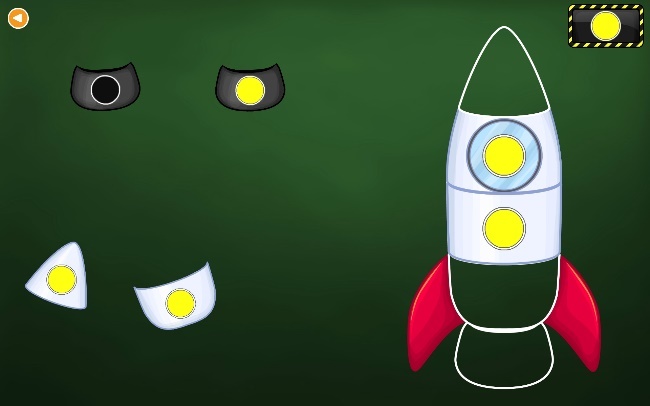 | | Planning, inhibition, visual search, sorting |
| Collecting nuts (Colours) | 2 | | In this app, a squirrel runs along a predetermined path to a destination and collects nuts, that are of different colours. Children are asked to collect only nuts in certain colours and bring them to the end destination on the board. | | 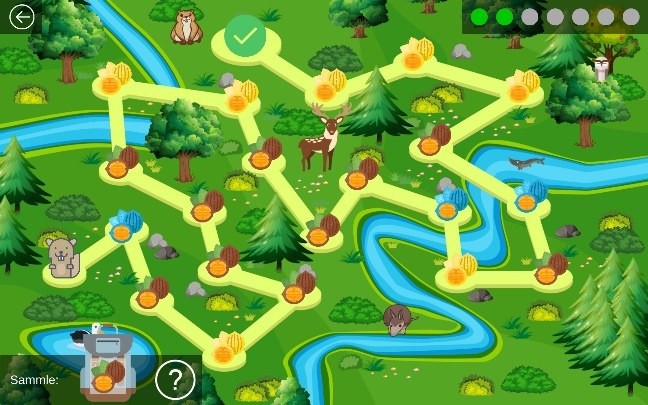 | | Planning, inhibition, visual search |

| **App Name** | | **Month** | | **Description – Control Apps** | | **Screenshot** | **Trained Skills** |
| --- | --- | --- | --- | --- | --- | --- | --- |
| Snakes & Ladders (Colours) | 2 | | This game is similar with the traditional Snakes & Ladders game except the board and the dice are marked with colours. This game can be played in multi-player mode. The colours are named during game play | | 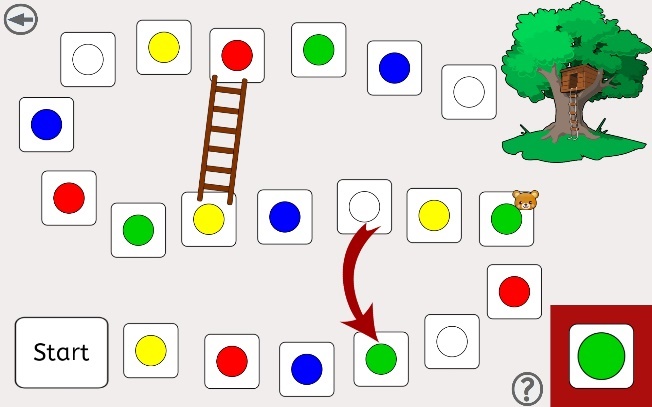 | | Colour recognition |
| Tap it! Animals | 2 | | In this app, animals come out of the ground rapidly and children are expected to tap on them with their fingertips as fast as possible before they disappear to collect points. | | 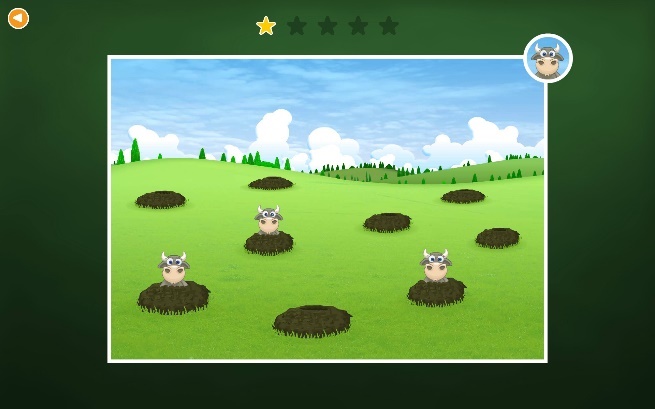 | | Processing speed, visual search, concentration |
| Sagomini Forest Flyer^1^ | 3 | | In this app, children are expected to navigate a flying bird in a forest and explore hidden tasks. | | 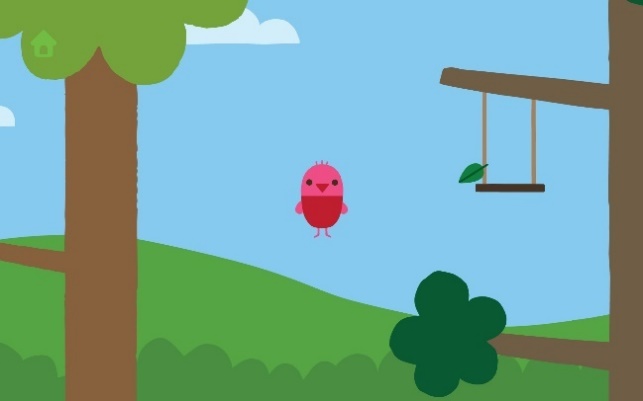 | | Navigation, hand-eye coordination |

| **App Name** | | **Month** | | **Description – Control Apps** | | **Screenshot** | **Trained Skills** |
| --- | --- | --- | --- | --- | --- | --- | --- |
| Sagomini Friends^1^ | 3 | | In this app, children (through an avatar) complete several tasks together with another avatar. These avatars collaborate and play together. | | 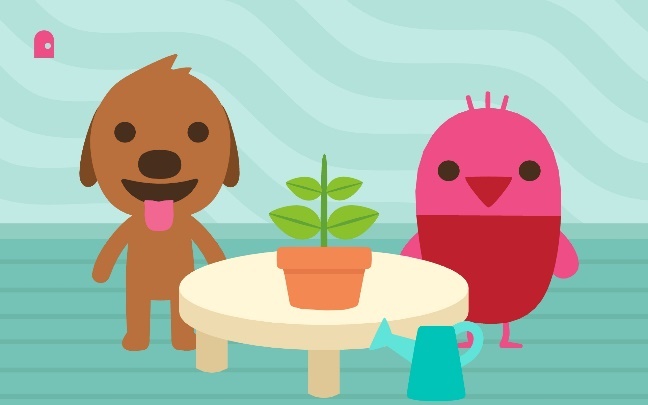 | | Socio-emotional skills, prosocial behaviour |
| Finding pairs (colours) | 3 | | In this app, one target colour is presented above the screen with a couple of objects to choose below it. The aim is to decide which objects’ colour is matching with the ‘target’ colour that is presented above. | | 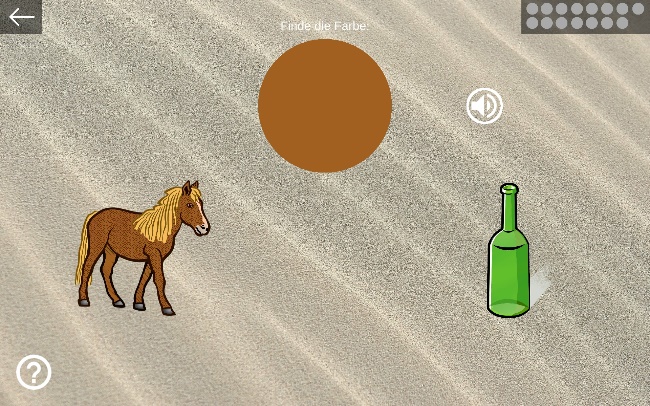 | | Colour recognition, inhibition |
| Bird Tower^1^ | 3 | | In this app, the children help a flappy bird trying to fly high as possible and catch the rewards, but also to avoid traps at the same time and try for a higher score each time. | | 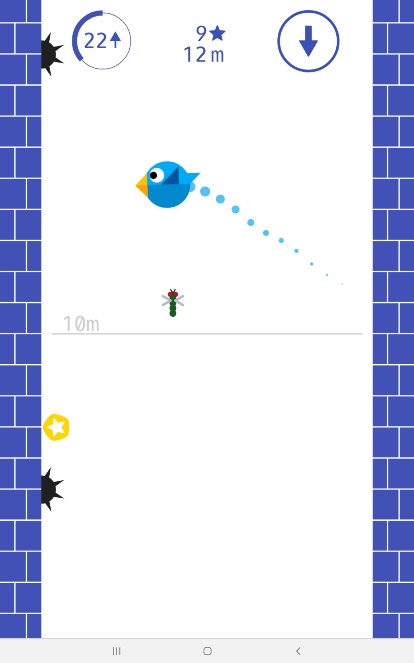 | | Hand-eye coordination, inhibition |

| **App Name** | | **Month** | | **Description – Control Apps** | | **Screenshot** | **Trained Skills** |
| --- | --- | --- | --- | --- | --- | --- | --- |
| Fish-maze^1^ | 4 | | In this app, children are expected to turn around a maze to help a fish to find its way out of it. | | 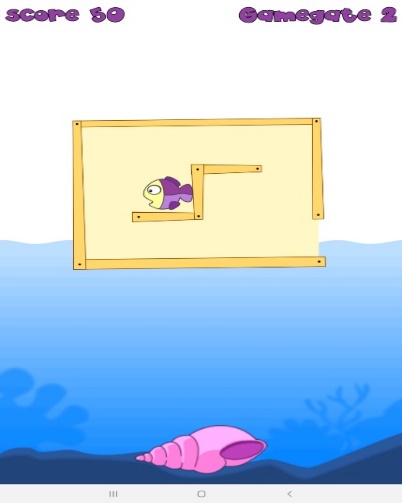 | | Hand-eye coordination |
| Colour-domino | 4 | | This game corresponds with the traditional analogue domino game. Here, children are expected to match the colours of two dots and to complete the row. | | 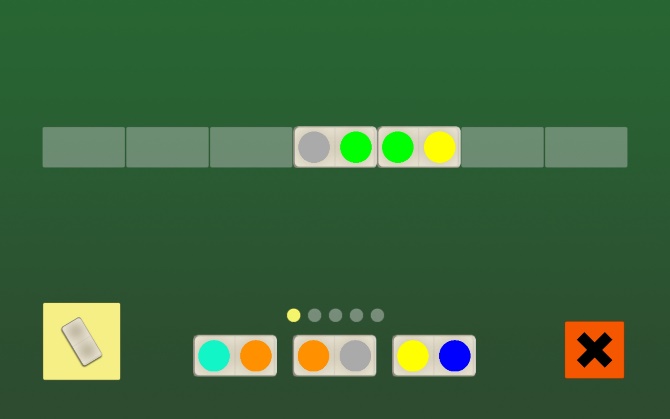 | | Colour recognition, logical thinking, decision making |
| Connect the colour dots | 4 | | In this app, children are asked to connect a duck with its egg, by drawing a line through the correctly coloured dots. | | 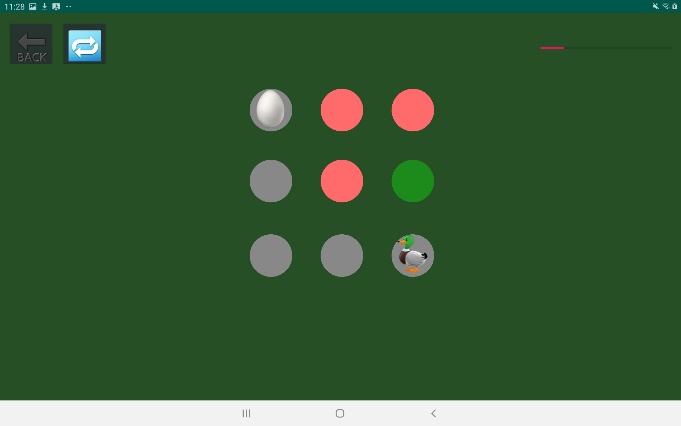 | | Hand-eye coordination, concentration |

| **App Name** | | **Month** | | **Description – Control Apps** | | **Screenshot** | **Trained Skills** |
| --- | --- | --- | --- | --- | --- | --- | --- |
| Animal maze^1^ | 5 | | In this puzzle app, children are expected to move an animal from a start point of a maze to the end point to let the animal free. | | 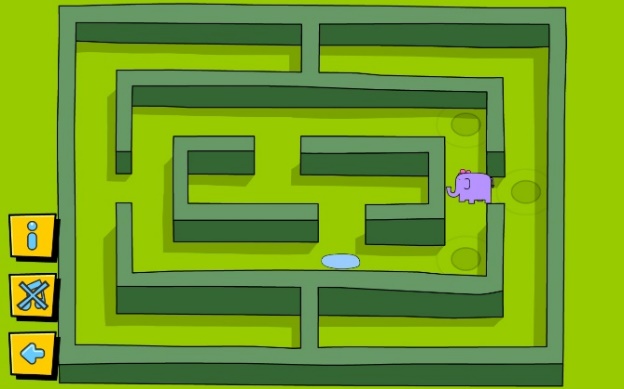 | | Hand-eye coordination, planning, concentration |
| Piano^1^ | 5 | | This app provides a virtual representation of a piano and allows children to learn and have fun with piano sounds. | | 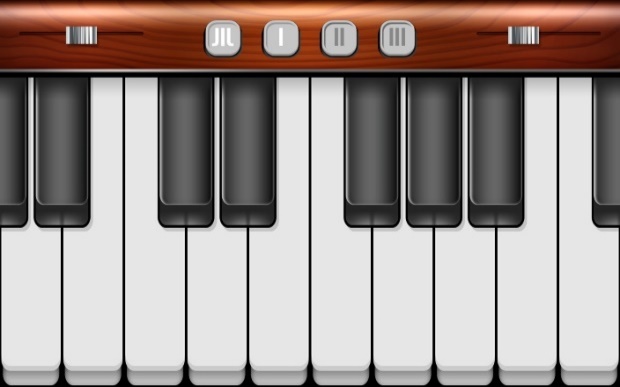 | | Musical learning, hand-eye coordination |
| Categorize colours | 5 | | In this app, children are expected to match objects with a given target box based on their colour. | | 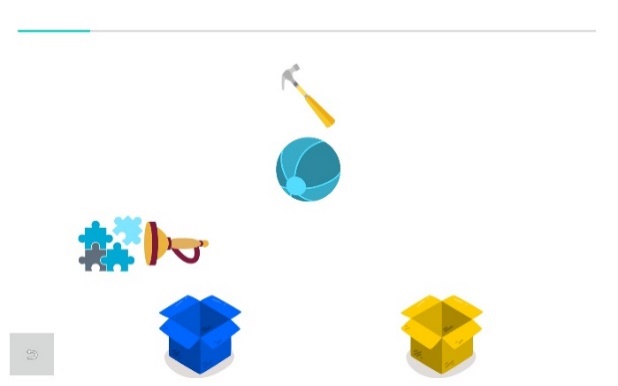 | | Colour recognition |

*Note*. ^1^The word puzzle app, Mathemarmite, Sagomini Forest Flyer, Sagomini Friends, Bird Tower, Fish-maze, Animal Maze, and Piano were not designed and developed within the framework of the Learning4Kids project.

**Figure S1**

*Change in the Numeracy Score across the Intervention Period for the Numeracy Intervention, the Literacy Intervention, the Tablet-Control, and the Control Group*
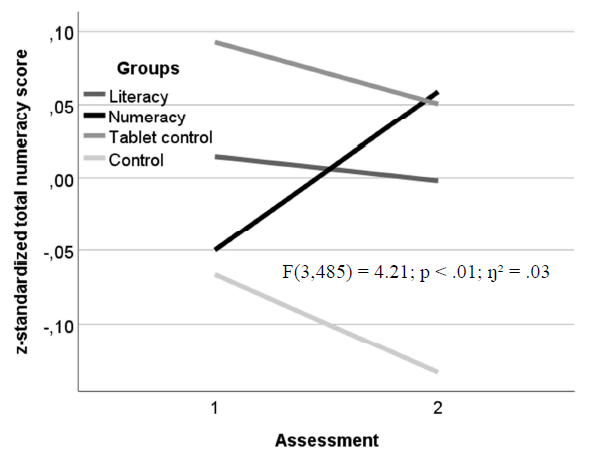


*Note.* Standardized scores were analysed and a decline does not indicate lower numeracy scores at t2 compared to t1. Controlled for child age, sex, and intelligence and family migration background and socio-economic status.

**Figure S2**

*Change in the Literacy Score across the Intervention Period for the Literacy Intervention, the Numeracy Intervention, the Tablet-Control, and the Control Group*

*Note.* Standardized scores were analysed and a decline does not indicate lower literacy scores at t2 compared to t1. Controlled for child age, sex, and intelligence and family migration background and socio-economic status.

**Figure S3**

*Change in the Numeracy Score across the Intervention Period for the Numeracy Intervention, the Tablet-Control, and the Control Group in Cohort 1 only*


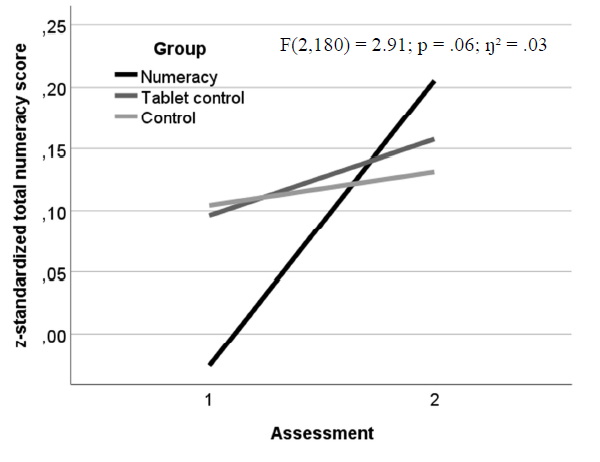


*Note.* Standardized scores were analysed and a decline does not indicate lower numeracy scores at t2 compared to t1. Controlled for child age, sex, and intelligence and family migration background and socio-economic status.

Figure S4

*Change in the Literacy Score across the Intervention Period for the Literacy Intervention, the Tablet-Control, and the Control Group in Cohort 1 only*


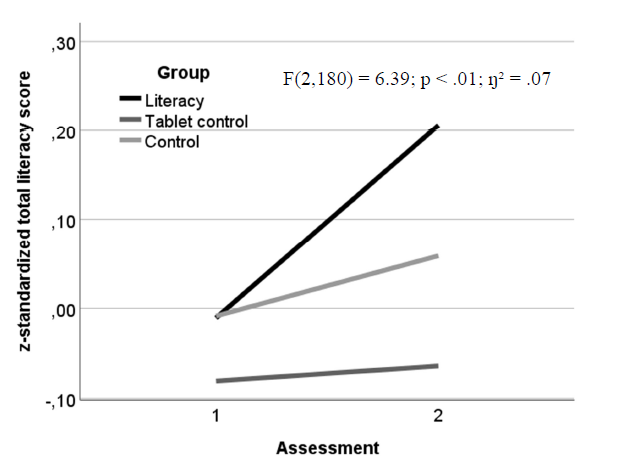


*Note.* Standardized scores were analysed and a decline does not indicate lower literacy scores at t2 compared to t1. Controlled for child age, sex, and intelligence and family migration background and socio-economic status.

Figure S5

*Change in the Numeracy Score across the Intervention Period for the Numeracy Intervention, the Tablet-Control, and the Control Group in Cohort 2 only*

*Note.* Standardized scores were analysed and a decline does not indicate lower numeracy scores at t2 compared to t1. Controlled for child age, sex, and intelligence and family migration background and socio-economic status.

Figure S6

*Change in the Literacy Score across the Intervention Period for the Literacy Intervention, the Tablet-Control, and the Control Group in Cohort 2 only*

*Note.* Standardized scores were analysed and a decline does not indicate lower literacy scores at t2 compared to t1. Controlled for child age, sex, and intelligence and family migration background and socio-economic status.
