# Supplementary material for: Learning apps at home prepare children for school
Source: Child Dev. 2024 Oct 24;96(2):577–90. doi: 10.1111/cdev.14184 (PMC11868692; doi:10.1111/cdev.14184)
Supplement: Supplementary file 1 — Appendix S1. [file CDEV-96-577-s001.zip › cdev14184-sup-0002-TableS4.docx]

**Table S4**

*Correlations of all Study Variables* (will be available as online excel file)
